# *UniversitätsSpital Zürich*: 80 years of neurosurgical patient care in Switzerland

**DOI:** 10.1007/s00701-017-3357-z

**Published:** 2017-11-13

**Authors:** Martin N. Stienen, Carlo Serra, Lennart H. Stieglitz, Niklaus Krayenbühl, Oliver Bozinov, Luca Regli

**Affiliations:** Department of Neurosurgery, University Hospital Zurich, Frauenklinikstrasse 10, 8091 Zürich, Switzerland

**Keywords:** History, Neurosurgery, UniversitätsSpital, Zürich, Zurich, Krayenbühl, Yasargil, Yonekawa, Regli, Brain surgery, Spine surgery, Europe, Anniversary

## Abstract

**Background:**

The predecessor of today’s Department of Neurosurgery, *UniversitätsSpital Zürich* (USZ), was founded 80 years ago as the first independent Swiss clinic dedicated to neurosurgical patient care. On the occasion of this anniversary, we aimed to highlight the history of neurosurgery as a specialty at the USZ, and to put it into the historical context of Swiss and European Neurosurgery.

**Method:**

A literature review was conducted and we searched the archives of the USZ and the city of Zurich, as well as those of Swiss journals to extract relevant published articles, books, historical reports and pictures. The USZ Department of Medical History, the Museum of Medical History and the Swiss National Library were contacted to provide source material. To further verify the content, (emeritus) faculty from the USZ and external experts on the history of Swiss neurosurgery reviewed the manuscript.

**Results:**

Surgeries of the head and spine had occasionally been conducted in Zurich by the general surgeons, Rudolf Ulrich Krönlein and Paul Clairmont, before an independent neurosurgical clinic was founded by Hugo Krayenbühl on 6 July 1937. This was the first Swiss department dedicated to neurosurgical patient care. Besides providing high-quality medicine for both the local and wider population, the department was chaired by eminent leaders of neurosurgery, who influenced the scientific and clinical neurosurgery of their time. As such, it has long been regarded as one of the top teaching and research hospitals in Switzerland and in Europe.

**Conclusions:**

On the occasion of its 80th anniversary, we have performed an in-depth review of its development, successes and challenges, with a special focus on the early decades. Reflecting on the past, we have identified common denominators of success in neurosurgery that remain valid today.

**Electronic supplementary material:**

The online version of this article (10.1007/s00701-017-3357-z) contains supplementary material, which is available to authorised users.

## Introduction

The advent of neurological surgery in Switzerland was undoubtedly due to Hugo Krayenbühl (born 3 December 1902 in Zihlschlacht; died 9 January 1985 in Zurich) [72]. The predecessor of today’s Department of Neurosurgery *UniversitätsSpital Zürich* (USZ) was founded 80 years ago, on 6 July 1937, by Professor Hugo Krayenbühl, as the first independent Swiss clinic dedicated to neurosurgical patient care. Additional specialised neurosurgical units were later established at the four other Swiss University Hospitals in Basel (Wilhelm Driesen, 1952), Bern (Hans Markwalder, 1953), Geneva (Aloys Werner, 1956), and Lausanne (Eric Zander, 1959), and at non-university hospitals in Chur (Charles Probst, 1967), St. Gallen (Gerhard Weber, 1970), Aarau (Charles Probst, 1973) and Lugano (Rezio Renella, 1989). Besides providing high-quality medicine for both the local and wider population, the department was chaired by eminent leaders of neurosurgery who strongly influenced the scientific and clinical neurosurgery of their time (Tables 1 & 2). As we shall see, several chairmen have played decisive roles in the development of microsurgical techniques in neurosurgery; techniques that have revolutionised modern neurosurgery and are today applied worldwide.

**Table 1:** Heads of the Department of USZ Neurosurgery (1937–2017)

Year appointed	Neurosurgeon
1937	Hugo Krayenbühl
1973	M. Gazi Yaşargil
1993	Yasuhiro Yonekawa
2007	Helmut Bertalanffy
2012	Luca Regli

**Table 2:** Timeline for neurosurgery performed in Zürich and at USZ

Date	Event
1882	The first article is published, giving evidence that neurosurgical procedures were carried out in Zürich by Rudolf Ulrich Krönlein [37, 51].
1895	Krönlein performs the first craniotomy for a cerebral tumorous lesion in Zürich [37, 52].
15 February 1910	The neurologist Otto Veraguth and surgeon Hans Brun are among the first remove an intramedullary lesion (tuberculoma at the level C3/4) [39, 87].
17 October 1927	Veraguth and Brun describe one of the first surgical treatments of a herniated lumbar disc [39, 88].
6 July 1937	The 12-bed department of neurosurgery at USZ (Klinik Hegibach) is founded by Hugo Krayenbühl.
13 July 1937	Krayenbühl introduces the diagnostic technique of ventriculography.
19 July 1937	Krayenbühl operates on the first patient with an intracranial tumour (37-year-old woman with sphenoid wing meningioma) after the foundation of a specialised department.
4 October 1938	Krayenbühl performs a third ventriculostomy in a patient with occlusive hydrocephalus due to a large midbrain tumour.
1938–1944	Krayenbühl introduces modern anaesthesiological techniques such as intratracheal anaesthesia.
1940	A carotid angiography is performed.
1948	Through cooperation with Rudolf M. Hess, electroencephalography (EEG) becomes available at USZ.
1951	The department is transferred to the new “Kantonsspital” building, comprising a 60-bed unit and two surgical theatres.
1953	Vertebral arteriography is performed. M. Gazi Yaşargil joins Krayenbühl’s department.
1955	Krayenbühl becomes a founding member and the first president of the Swiss Society of Neurosurgery.
1957	Stereotaxy is introduced and increasingly performed by Krayenbühl, Yaşargil, and their co-workers.
1959	The first European Congress of Neurosurgery is held in Zürich.
1960	Yaşargil performs a Th11 and 12 corporectomy and implants a specially designed telescopic screw device through a trans-thoracic approach [78].
1963	A surgical microscope is purchased (binocular Zeiss).
1966	It is reported that from 1966 Krayenbühl used neuropsychological tests on a regular basis in order to assess patients.
1967	Microneurosurgery is used on a regular basis after Yaşargil’s return from Raymond M. P. Donaghy’s department in Burlington, Vermont (USA).
30 October 1967	Yaşargil performs the first EC-IC bypass in a patient with Marfan’s syndrome and complete occlusion of the MCA.
1972	Krayenbühl retires, leaving behind a 73-bed unit with a team of 16 neurosurgeons treating about 2,000 patients annually. Yaşargil becomes the new Chair of the department.
1973	Yaşargil performs the first trans-sylvian selective amygdalohippocampectomy for medically refractory epilepsy.
April 1977	The USZ is the second public hospital after the University hospital Basel to purchase a CT scanner enabling head scans [73].
1983	The foramen ovale (FO) electrode, a semi-invasive method for preoperative assessment of limbic epilepsy, is developed [26, 102].
1983	Routine MRI becomes available at USZ for neurosurgical patients.
1986	Functional imaging by single-photon emission computerised tomography (SPECT) and positron emission tomography (PET) is made available at USZ for neurosurgical patients.
1989	The introduction of the selective and superselective amytal memory test allows for more exact presurgical language and memory investigations (Wada test) and better selection of surgical candidates.
1993	Yaşargil retires and Yasuhiro Yonekawa becomes the new head of the department.
1995	The USZ acquires the first General Electric open intraoperative low-field MRI machine in Switzerland [and second in the world after Brigham Hospital in Boston, MA (USA)].
1998	A neurosurgical intensive care unit is established, led by Emanuela Keller.
2001	The supracerebellar transtentorial approach to the posterior temporomedial structures is proposed by Yonekawa.
2007	Yonekawa retires and Helmut Bertalanffy becomes the new head of the department.
2009	The first report of transcranial MR-guided high-intensity focused ultrasound surgery (tcMRgHIFUS) is published.
2010	René Bernays becomes interim chief of the department.
2012	Luca Regli becomes the new head of the department.
2013	Installation of an intraoperative high-field MRI machine.
2013	A prospective patient registry is installed to assess quality of care, outcomes and complications.
2013	Three-dimensional endoscopy is available at USZ.
2015	Intraoperative CT imaging is available at USZ.

**Fig. 1:**
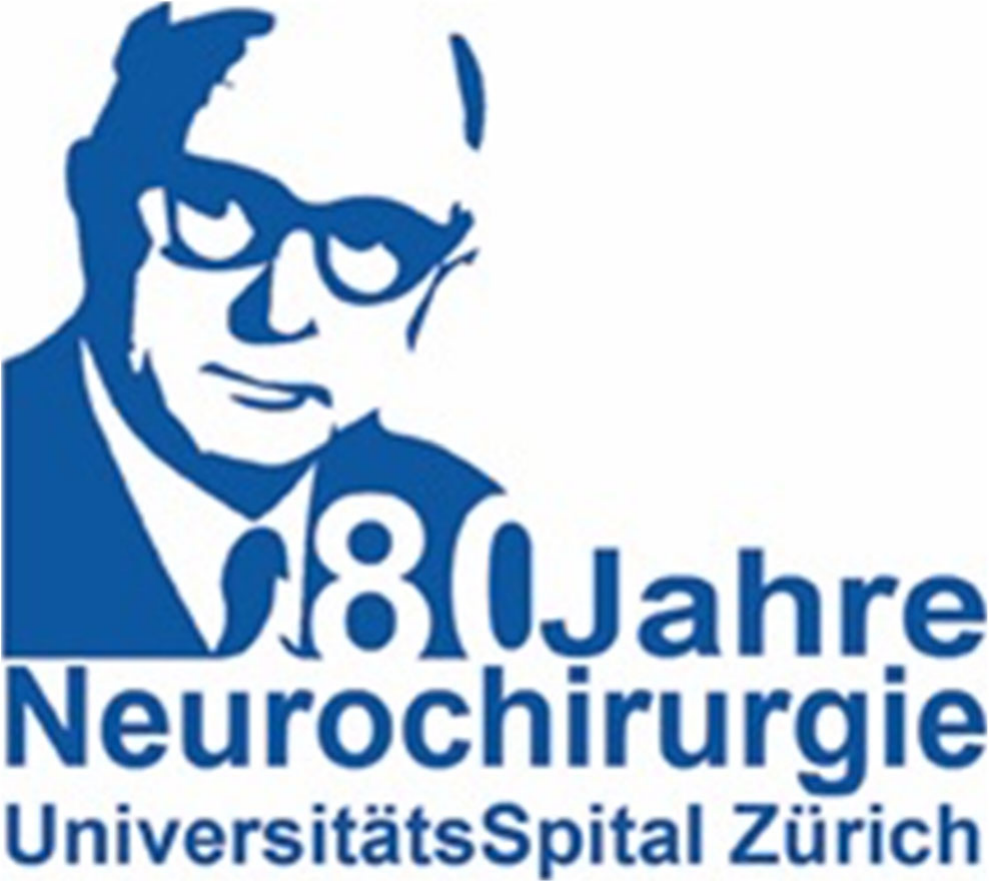
Eightieth anniversary logo of the *UniversitätsSpital Zürich* (USZ)

Since its foundation, the USZ has been regarded as one of the top neurosurgical teaching and research hospitals in Switzerland and in Europe. On the occasion of its 80th anniversary (Fig. 1), this article highlights the early development of the department and the challenges it faced, and places them into the historical context of European neurosurgery. Special emphasis is placed on the founding era of Hugo Krayenbühl.

## Methods

A literature review was conducted using MEDLINE, Embase, Science Direct, and Google Scholar. We also searched the archives of the USZ and the city of Zurich, as well as those of Swiss journals (Swiss archives of Neurology, Psychiatry and Psychotherapy; *Revue Médicale Suisse*; *Praxis*; *Correspondenz-Blatt für Schweizer Aerzte*) to extract relevant published articles, books, historical reports and pictures. The USZ Department of Medical History, the *Medizinhistorisches Museum Zürich*, and the Swiss National Library (*Nationalbibliothek*) were contacted to provide source material. To further verify the content, (emeritus) faculty from the USZ and external experts on the history of Swiss neurosurgery (mentioned in the acknowledgements) reviewed the manuscript.

For reasons of simplicity, the name of today’s USZ, formerly the *Hegibach* ward of the surgical clinic (before 1952), the *Kantonsspital* in Zurich (1952–1977), and the University Hospital Zurich (since 1978), is homogenously referred to as the USZ throughout this article.

## Neurosurgical care in Switzerland before the founding of a distinct specialty

In the Western world, modern neurosurgery began its rise in the late nineteenth century and the beginning of the twentieth century. At that time, selected neurosurgical procedures were usually performed by general surgeons. In Zurich, Rudolf Ulrich Krönlein (1847–1910) was the chief surgeon at the USZ from 1881 to 1910 [36, 37]. The earliest published neurosurgical work at the USZ dates back to 1882. This work demonstrates that Krönlein supported the surgical treatment of impressed skull fractures; in particular, in patients with signs of elevated intracranial pressure (ICP) [50]. Relating his clinical observations to modern theories of cerebral topography, as proposed by Fritsch and Hitzig, Ferrier, and others of that time [90], Krönlein was among the first to reconfirm the association of Rolando’s cortical area with motor function in humans. In 1895, he reviewed the literature up to 1894 and noticed a rapid increase in brain tumour surgery, from only 15 patients before 1890, to 67 patients between the years of 1890 and 1894, despite a high mortality rate of nearly 30% [51]. For surgery on lesions that were either not found or could not be completely resected, mortality was as high as 75%, and he concluded that only a small percentage of brain tumours could be cured surgically at that time [51]. In the same year, he performed the first trepanation for a cerebral tumorous lesion (tuberculoma). The surgery under ether anaesthesia took 1 h, and the patient was reported to have lived 10 years post-surgery [36]. Krönlein’s important contributions to neurosurgery have recently been summarised elsewhere, and it is beyond the scope of this article to go into further detail on this topic [36].

About 100 km west of Zurich, the 1909 Nobel Prize laureate Emil Theodor Kocher (1841–1917) served as chief surgeon at the university hospital, the *Inselspital* in Bern. His main neurosurgical interests were in epilepsy surgery [88], the surgical therapy of spinal trauma and brain tumours, as well as theories on ICP and cerebral blood flow (CBF) [27, 37, 39, 88]. As early as 1887, he reported having performed an osteoplastic trepanation of the left occipital lobe to resect a 2-cm brain tumour after transcortical exposure in a patient presenting up to 30 epileptic seizures per month [44]. Kocher was the first surgeon in a Swiss clinic to perform transnasal surgery for a pituitary tumour in a patient with acromegaly (1909) [27, 39], only 2 years after Hermann Schloffer in Innsbruck, Austria [55]. Kocher was also among the first to surgically treat trauma and diseases of the spine and spinal cord (from 1872 onwards), as evidenced from his 245-page publication on this topic [45, 46]. The early illustration of a dermatome chart in his publication has recently been highlighted [39]. Together with Ernst von Bergmann (1836–1907, Berlin), Krönlein and Kocher built a triumvirate of academic pioneers with similar intentions and priorities for the emerging field of neurosurgery in the German-speaking countries. However, the establishment of neurosurgery as an independent discipline was inconceivable at that time [37]. Moreover, Kocher thought that general surgery could adequately cover the field of neurological surgery if neurologists, respectively internists, helped with the diagnostic part:

*Wo Internisten und Chirurgen einander in die Hände arbeiten, wo jeder Gehirnfall von vornherein von beiden angesehen und beurteilt wird, da lässt sich Grosses erreichen.* (Cited after [99])

(Where internists and surgeons work hand in hand, where neurological emergencies are a priori evaluated by both, great [results] can be achieved.)

At the USZ, the head of surgery, Paul Clairmont (born 10 January 1875; died 1 January 1 1942), performed trepanations in cases of traumatic brain injury (TBI) and suspected intracranial haemorrhage. In 1923, he reviewed the 275 patients with TBI from his clinic between 1918 and 1922. In his article, he described the surgical technique of trepanation and reported on the value of lumbar puncture as a diagnostic and therapeutic tool in TBI [18]. The Zurich neurologist Otto Veraguth reported one of the first successful surgical treatments of a lumbar disc herniation {performed in 1927 by a surgeon from Lucerne, Hans Brun (1874–1946). [38]}. This happened well before Dandy, or Mixter and Barr’s so-called landmark paper of 1934 [86]. Both Veraguth and Brun proved to be pioneers in regards to the surgical treatment of intramedullary spinal lesions: in 1910, Hans Brun became the third surgeon (after Anton von Eiselsberg in Vienna and Charles Elsberg in New York) to successfully remove an intramedullary lesion in a patient [38, 85].

In the French-speaking part of Switzerland, first reports include surgery on a pituitary tumour using a transnasal approach, performed by the general surgeon Albert Jentzer (Geneva) in 1924. Jentzer also designed a trephine for craniotomies, which was incorporated into the medical instrument set of the Swiss Army [72]. Likewise, François Ody, from the surgical department in Geneva, reported on attempted suboccipital decompression procedures for patients with traumatic oedema [65]. He is likewise known for an adventurous emergency operation performed on a friend with a perforating cranio-cerebral injury, after an accident at 9,200 ft (3,051 m) in the refuge *des Grands Mulets* in the Mt. Blanc range, where he used forks, his pocket knife and handkerchiefs for a temporary decompression ([66] cited by [72]).

It was the US surgeon Harvey Cushing (1869–1939) who first established neurosurgery as a distinct speciality in the early 1900s [21]. In the following decades, many young surgeons travelled to his clinic at Harvard Medical School in Boston in order to be trained. One of his European pupils, Sir Hugh Cairns (1896–1952) from London, would be the future teacher of Hugo Krayenbühl.

## USZ neurosurgery under the direction of Hugo Krayenbühl (1937–1973)

**Fig. 2:**
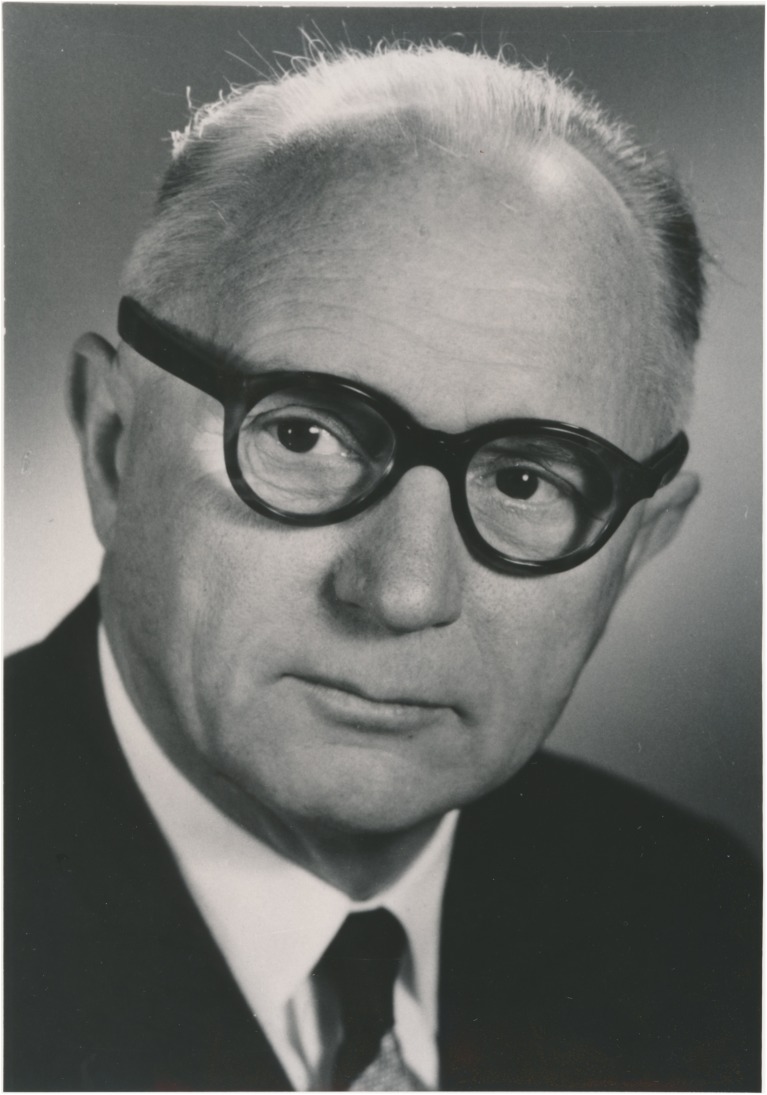
Portrait of Hugo Krayenbühl. Black/white (b/w) print in photo album. Photograph: Hans Peter Weber. Zurich, 1965. Photo credit: *Archiv für Medizingeschichte Universität Zürich (AfMZH) IN 37.02.01*. Published with permission

**Fig. 3:**
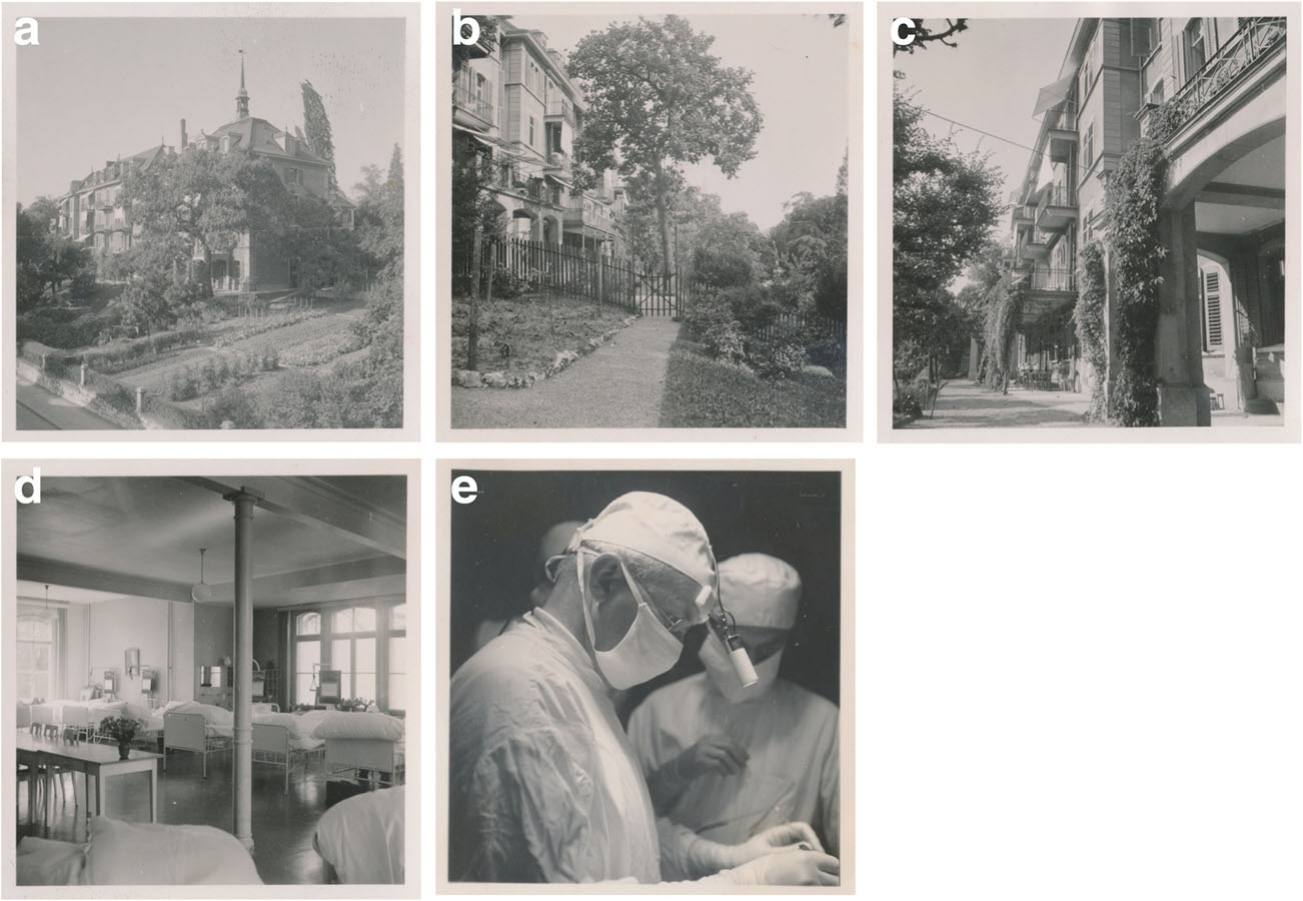
The first department of neurosurgery was situated in the *Aussenstation Hegibach* from 1937 to 1951. **a** View from the Heliosstrasse. **b** Outside view with garden. **c** Outside view near entrance. **d** Inside view depicting the patient dormitory. **e** Hugo Krayenbühl and Gerhard Weber operating. Series of eight b/w prints on carton (only five shown). *Pressedokumentation*, photographer unknown. Zurich, 1949. Photo credit: *Archiv für Medizingeschichte Universität Zürich (AfMZH) PS_gf IN 37.01:002*. Published with permission

**Fig. 4:**
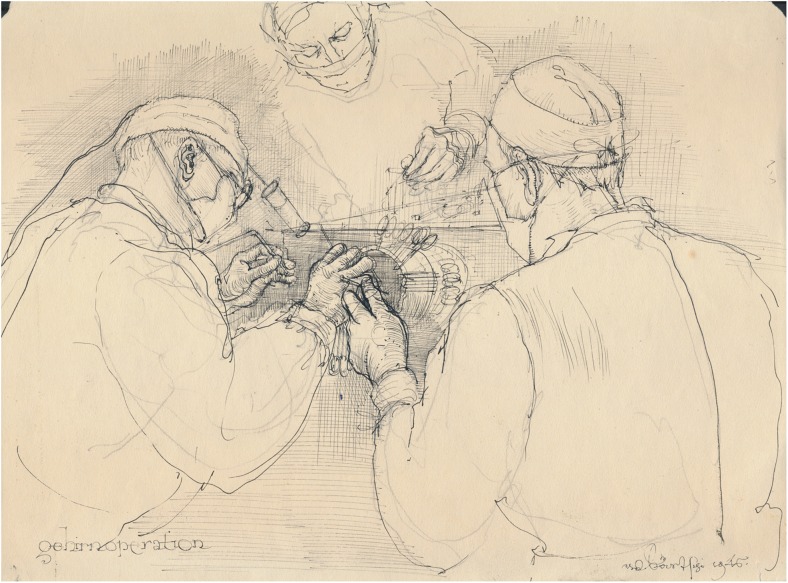
Hugo Krayenbühl operates on the brain (*gehirnoperation* = brain surgery), assisted by two colleagues. For illumination, he often wore a headlamp with power connection, springy metal-buckle and padding, that he had brought from London. Pencil and ink painting. Artist: Werner Bärtschi, 1946. Photo credit: *Archiv für Medizingeschichte Universität Zürich (AfMZH)* Bsc 7–1. Published with permission

**Fig. 5:**
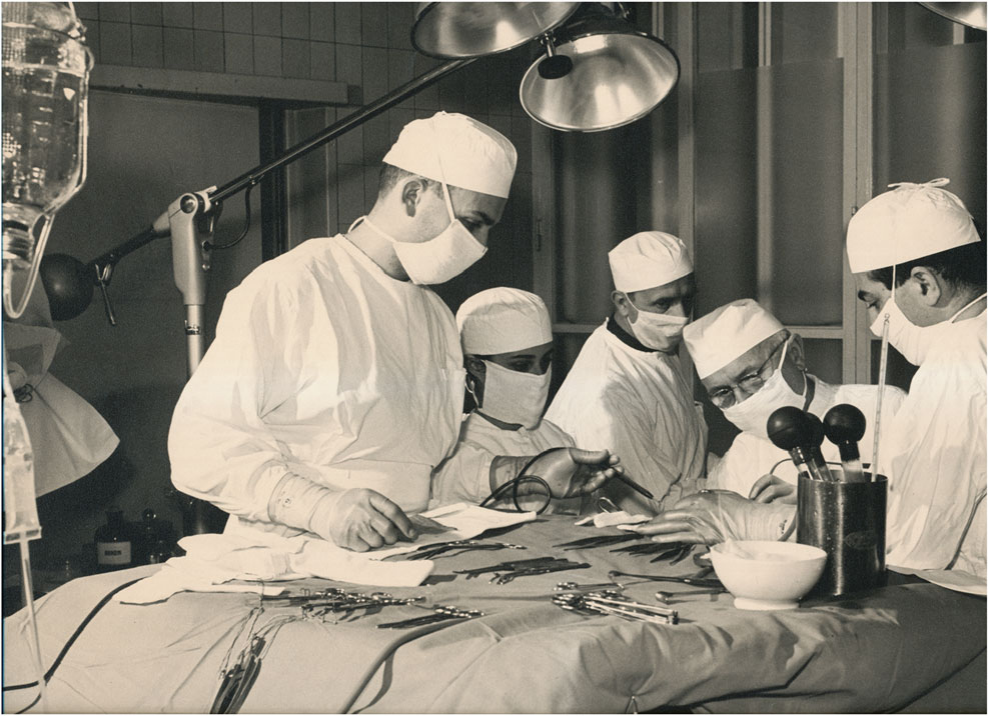
Hugo Krayenbühl (second from the right, wearing glasses) operating. B/w print on carton. Photographer: Hans Peter Weber. Neurochirurgie Kantonsspital, 1970s. Photo credit: *Archiv für Medizingeschichte Universität Zürich (AfMZH)* PS_gf PN 222. Published with permission

**Fig. 6:**
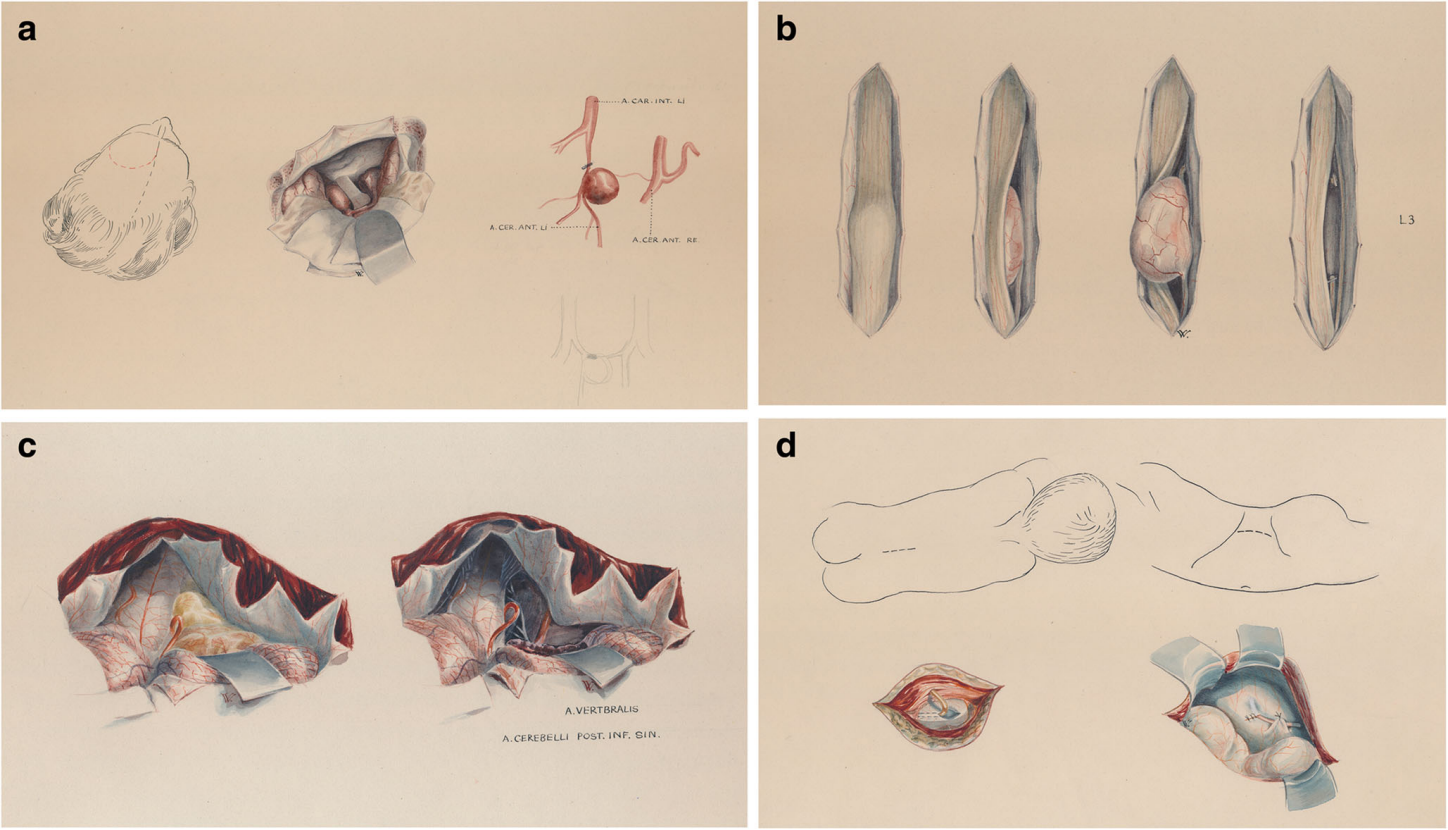
Examples of the illustrations that often accompanied surgical procedures. Patient name, disease, and date of surgery are given on the top of each figure (not shown). Illustrator: Hans Peter Weber. Image credit: *Archiv für Medizingeschichte Universität Zürich (AfMZH)*. Published with permission. **a** Case of a 57-year-old woman with an anterior communicating artery aneurysm (surgery on 25 August 1950). PN 222.02.01-1a. **b** Case of a 44-year-old man with a neurinoma between L2-L4 (surgery on 26 September 1950). PN 222.02.01-1c. **c** Case of a 35-year-old male with an ependymoma exiting through the left of the foramen of Luschkae (surgery on 8 May 1953). PN 222.02.01-2a. **d** Case of a 3 month-old boy with “hydrocephalus internus communicans congenitalis” receiving a lumbo- peritoneal anastomosis for cerebrospinal fluid diversion (surgery on 11 March 1953). PN 222.02.01-2d

Hugo Krayenbühl (Fig. 2; Supplementary Figs. S1 and S2) was the first neurosurgeon at the USZ. He had studied for over 15 years in Geneva, Zurich, Kiel, Paris, St. Gallen and Berlin. On his return from working with Hugh Cairns, with whom he had spent two additional years as a graduate student in London and maintained a life-long friendship (Supplementary Fig. S3), he performed six major neurosurgical operations at the end of 1936 (on four patients with brain tumours, one patient with trigeminal neuralgia and one with a traumatic intracranial haemorrhage) [99]. Krayenbühl set up the new 12-bed neurosurgical clinic (Klinik Hegibach, Heliosstrasse, Zürich; Fig. 3) on 6 July 1937, under the patronage of his chief, the Professor of Surgery, Paul Clairmont, and injecting personal funds (Fig. 3a–d) [33, 99, 113]. Krayenbühl provided surgical instruments that he had bought in London, such as a surgical headlamp (Figs. 3e and 4), retractor, tweezers, chisel, flexible spatulas, Pennybacker bone rongeur, nerve root hooks and angled scissors (Fig. 5). The Rockefeller foundation donated an X-ray machine. The first intervention was a ventriculography performed on a 34-year-old man on 13 July 1937. Krayenbühl operated on the first brain tumour patient with the independent unit (a 37-year-old woman with sphenoid ridge meningioma) on 19 July 1937. This intervention took almost 9 h. It was performed under local anaesthesia. The long-term follow-up indicated that the patient was in good health for 30 years [33]. By the end of 1937, a total of 60 patients had been hospitalised in the new neurosurgical unit, including 25 patients with brain tumours, of which 18 were treated surgically [99]. Lumbar disc surgery was also performed on a regular basis from 1938 [72]. From the 1940s onward and for operations in the prone position (posterior approach to the spine, cerebellar operations) in particular, Krayenbühl used the technique of endotracheal intubation. It is reported that he preferred to operate at night, as there were fewer distractions [33], and with the assistance of general surgeons or medical students that he selected during his lectures [99]. He demanded that at the time of surgery, all relevant patient information was summarised on a red piece of paper that he considered an essential part of the patient file, attesting to his well-organised character. The chosen surgical approach and the intraoperative findings were often documented by drawings (see below; Fig. 6a–d). Only recently have the hand-written notes of his *Operationsbuch* (= surgical records) been systematically reviewed (Supplementary Fig. S5) [33]. Supplementary Fig. S6 shows the rapid increase in the number of diagnostic and surgical interventions in the initial years between 1937 and 1945. Techniques enabling efficient and safe surgery were systematically introduced under his lead [72]: cerebral angiography from 1937, intratracheal anaesthesia with nitrous oxide, enallylpropymal (Narconumal) and ether between 1938 and 1944, electroencephalography in 1948, stereotaxy in 1957, neuropsychology in 1966 and microneurosurgery in 1967.

In 1941, Krayenbühl attained the academic rank of *Privatdozent* with a thesis on cerebral aneurysms (Supplementary Fig. S4) [47]. In 1948, he became *ausserordentlicher Professor* (= extraordinary professor). In 1951, the neurosurgical unit was transferred to the new *Kantonsspital* building, where Krayenbühl received a 60-bed unit and two operation theatres. Finally, in 1963, Krayenbühl was promoted as full professor in neurosurgery. At the time of his retirement in 1973, at the age of 71 (Supplementary Fig. S7), the neurosurgical department at the USZ consisted of 16 neurosurgeons, who treated 2,000 patients annually in a 73-bed unit [118].

**Fig. 7:**
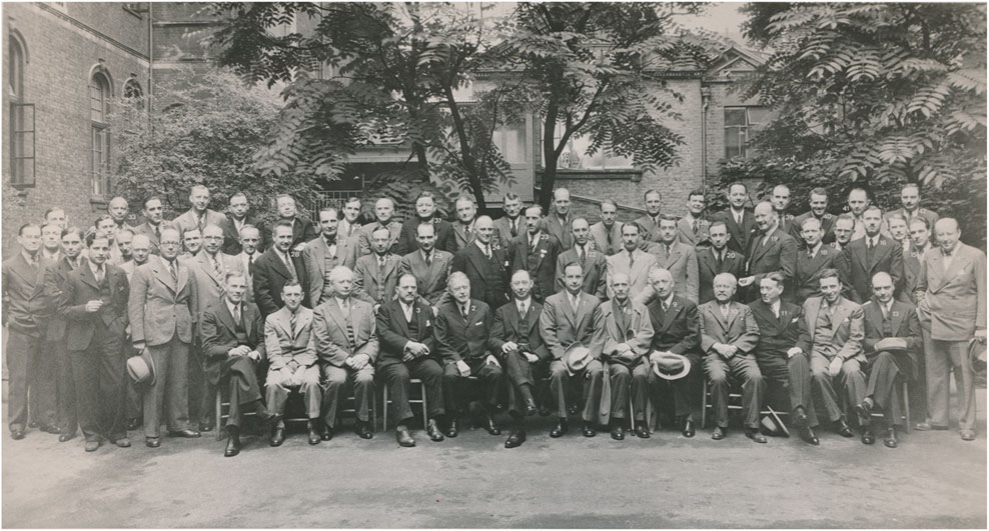
Attendees of the Second International Neurology Congress in London, August 1935. Special combined meeting with the American Neurosurgical Society, the Harvey Cushing Society and the Society of British Neurological Surgeons. Photo taken at the Garden of the National Hospital, Queen Square, depicting 43 participants and indicating names. Hugo Krayenbühl is second from right, in the back row (*41*). Among the participants were Geoffrey Jefferson (*1*), Charles Frazier (*2*), Clovis Vincent (*4*), Hugh Cairns (*6*), Otfried Förster (*8*), Thierry de Martel (*9*), Ludvig Puusepp (*10*) and Egas Moniz (*11*). B/w print in photo album. Photo credit: *Archiv für Medizingeschichte Universität Zürich (AfMZH) IN 37.02.01*. Published with permission

**Fig. 8:**
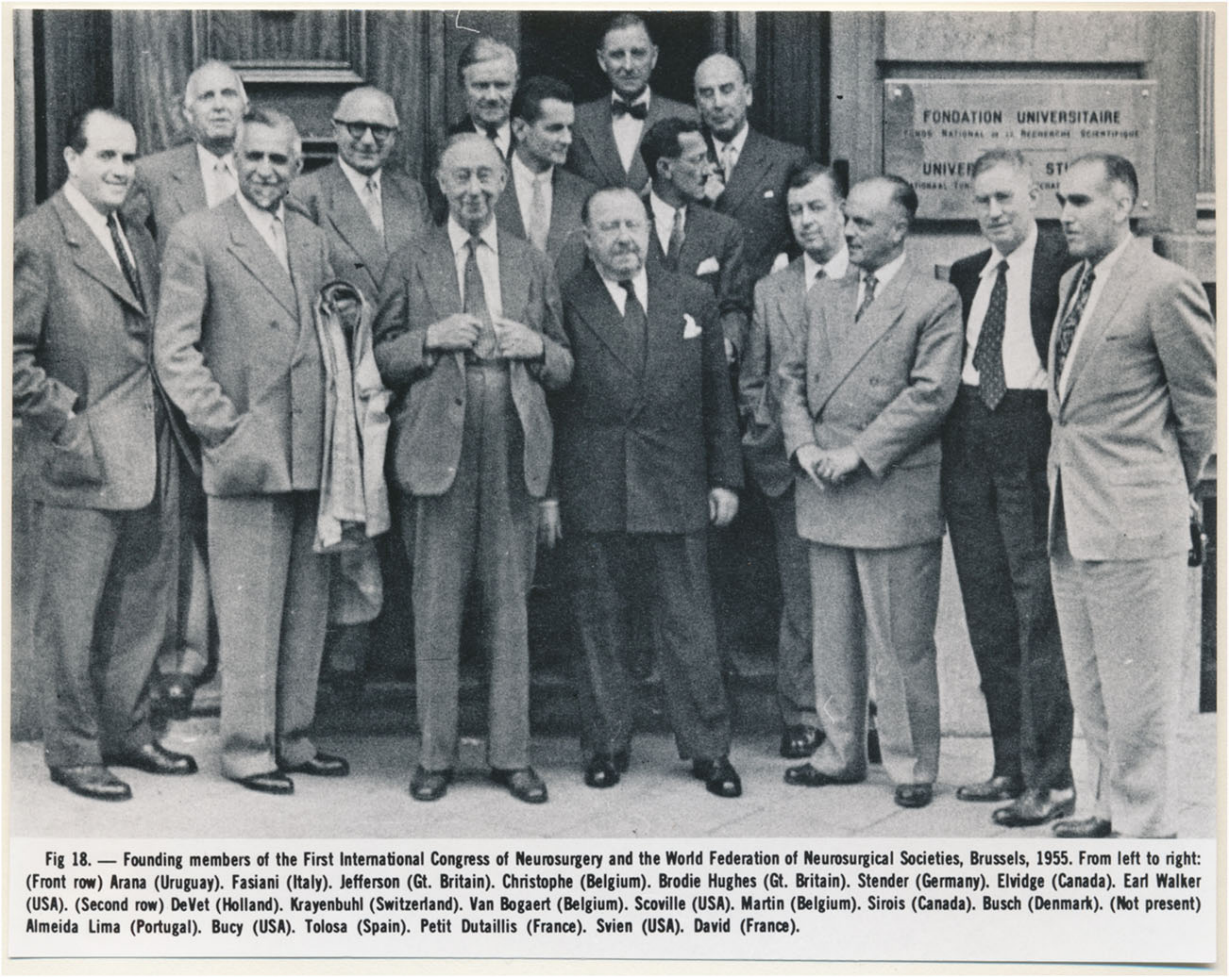
Founding Members of the First International Congress of Neurosurgery and the World Federation of Neurosurgical Societies, Brussels, 1955. Group photo depicting 15 participants with name index. Hugo Krayenbühl is third from the left, in the second row (round glasses). Photo credit: *Archiv für Medizingeschichte Universität Zürich (AfMZH) IN 37.02.01*. Published with permission

Krayenbühl has been described as one of the world’s leading physicians/neurosurgeons of his time, comparable to both Horsley and Cushing, by eminent colleagues such as Wilder Penfield of McGill University (Montreal), his contemporary Paul C. Bucy from Chicago, or his pupil and successor at the USZ, M. Gazi Yaşargil [16, 67, 105, 109]. His tenet was “*Der Neurochirurg ist ein Neurologe, der operieren kann*” (“A neurosurgeon is a neurologist who knows how to operate”) [3], indicating the importance he placed on solid neurological training, as Cushing did before him [99]. This firm grounding in neurology distinguished him from many of his general and neurosurgical contemporaries, and he earned the admiration of his fellow physicians for being both a superb clinical neurologist as well as an outstanding surgeon. He would actively seek contact with the leading neurologists and neurosurgeons of his time, as indicated by his attendance at international congresses (Figs. 7 and 8). In the USZ archive, for example, we found personal correspondence between Krayenbühl and Wilder Penfield dating from 1949, in which both exchange their ideas on how the perfect neurosurgical operating theatre should be designed (with hand-made illustrations) [68]. He not only founded a Swiss school of neurosurgery, but his followers were frequently elected chiefs of departments, such as in St. Gallen (Gerhard Weber), Lausanne (Eric Zander), and Geneva (Alois Werner) [8]. Until the early 1970s, it was a requirement for all Swiss neurosurgeons to have worked under Krayenbühl before they were authorised to practice in other clinics. Moreover, Swiss neurologists that would soon qualify for a position as the head of a department [e.g. Marco Mumenthaler (Bern), Franco Regli (Mainz and Lausanne)] were asked to spend 1 year in Krayenbühl’s clinic. These directives ensured a common level of qualification across Swiss clinical neuroscience.

Krayenbühl mastered multiple languages. This enabled him to forge and maintain international relations, and attracted many renowned neurosurgeons from Europe, the USA, and Japan to train under his supervision in Zürich [113]. He made neurosurgical education a main focus of his daily work. He emphasised not only professional expertise, but patient care with respect and dignity [99, 109]. He was reported to always have the well-being of his patients as his first priority, and to guide his employees through objective criticism and stimulating encouragement [3].

Krayenbühl was active in the World Federation of Neurosurgical Societies (WFNS) and was a founding member of the First International Congress of Neurosurgery organised by the WFNS in 1955 (Brussels; Fig. 8). He represented Switzerland and acted as their honorary president. He organised and hosted the first European Congress of Neurosurgery in Zurich (1959), which eventually led to the foundation of the European Association of Neurosurgical Societies (EANS) in 1971. He was among the founding members of the Swiss Neurosurgical Society, held the position of its first president from 1955 to 1961, and became its first honorary member in 1974. He was also the president of the *Societé de Neurochirurgie de Langue Française* and received honorary doctorates from the Universities of Lausanne and Geneva [105, 109]. At his retirement in 1973, Krayenbühl left a soundly based department filled with young people striving for leadership at the various growing points of the expanding field of neurosurgery.

## USZ neurosurgery under the direction of M. Gazi Yaşargil (1973–1993)

**Fig. 9:**
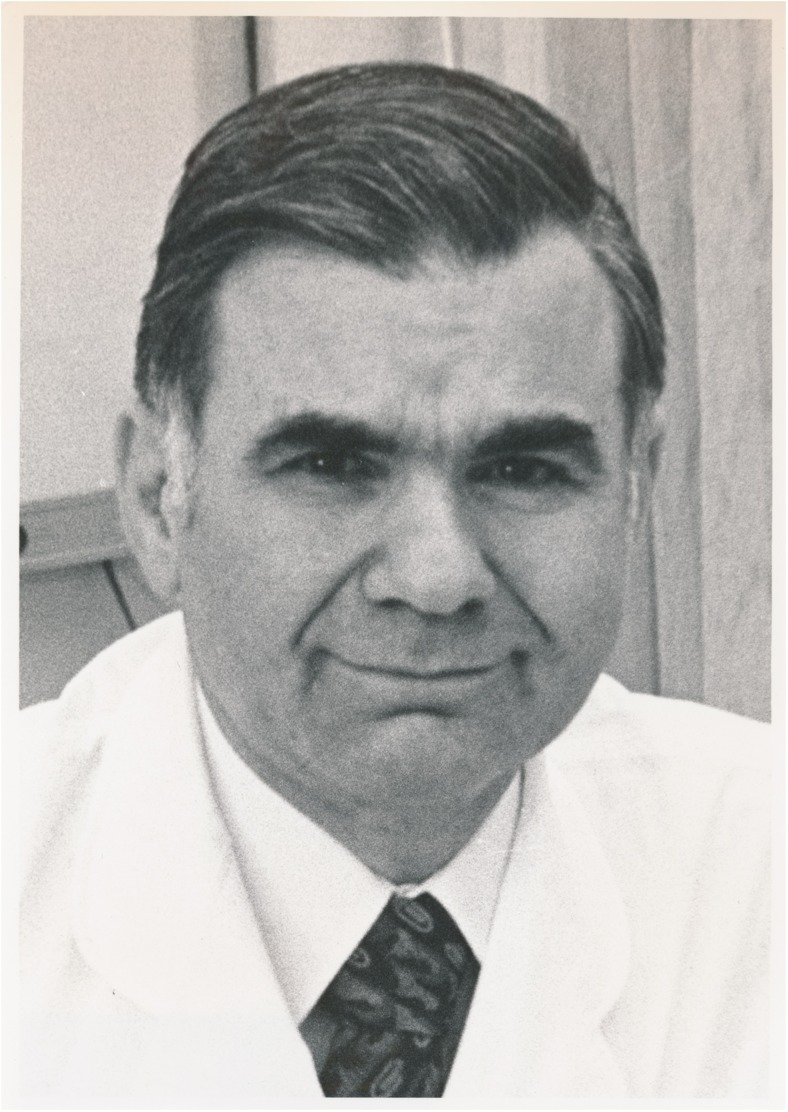
Portrait of M. Gazi Yaşargil. B/w print in photo album. Photograph: Hans Peter Weber. Zurich, 1973. Photo credit: *Archiv für Medizingeschichte Universität Zürich (AfMZH) IN 37.02.01*. Published with permission

M. Gazi Yaşargil (born 6 July 1925; Fig. 9) himself has summarised his early years and the inspiring encounters he had as a child and young adult, which paved the way for his profound interest in clinical neuroscience [106]. After an internship as a nursing aid in Naumburg an der Saale (Germany), he studied Medicine in Jena (Germany) during the Second World War. When the US and Russian troops made their approach, he was forced to leave Germany and continued his studies in Basel (Switzerland), where he had his first experience with microsurgery—in frogs [106]. After his board examination, he studied the anatomy of white matter tracts in Josef Klingler’s (1888–1963) anatomy laboratory and went on to pursue a residency in psychiatry, neurology, general surgery and internal medicine, in the Berne region, i.e. Interlaken and Münsingen.

He joined Krayenbühl’s department in 1953 and found in his chairman a teacher and mentor. Although he is more remembered for other achievements (see below), Yaşargil was an active spine surgeon who developed his own surgical instruments and materials, fulfilling the needs of each individual case. In 1960, for the removal of a giant cell tumour of the 11th and 12th thoracic vertebrae, he created a “telescoping screw” that he implanted using a trans-thoracic approach [76], and which seems to have inspired the creation of today’s frequently used expandable cages for traumatic and oncological indications. After professional visits to Traugott Riechert (Freiburg, Germany) and Jean Talairach (Paris, France), Yaşargil introduced stereotaxy to treat movement disorders and epilepsy at the USZ [8]. In the field of functional neurosurgery, he performed about 800 operations between 1958 and 1965, before concentrating on his main interest: the field of neurovascular surgery.

In 1963, interest in microsurgery was on the rise, and a binocular Zeiss microscope—an OPMI-1 on a NC-1-stand—was purchased. The first operations were performed to remove herniated disks. Unsatisfied with the balancing of that microscope, Yaşargil and collaborators from the Contraves company improved upon it by adopting the neutralisation of recoiling forces applied in canons (parallelogram-levers), and adding lighting, video and still photography, which were introduced in the legendary NC-1 model. From 1965 on, Yaşargil devoted himself to the development of microneurosurgery. After returning from a 2-year microsurgery fellowship in Raymond M. P. Donaghy’s (1919–1991) department in Burlington, Vermont (USA) [56], Yaşargil developed microsurgery with enthusiasm and strengthened the role of Zürich in the expansion of the field. Important concepts largely developed by Yaşargil include the combination of microsurgical techniques with bipolar coagulation—originally invented by Leonard Malis (1919–2005) [57]—the concept of arachnoidal exploration along cisterns as natural pathways, and the concept of the segmental and compartmental occurrence of vascular and neoplastic lesions of the central nervous system (CNS) with their predilection sites. These achievements allowed microsurgery to gradually mature and progress [106]. Both junior and senior neurosurgeons from all over the world travelled to Zürich in order to see, learn and acquire microsurgical techniques, applied under 4- to 25-fold magnification, improved lighting, and using special micro-instruments. Likewise, patients from near and far sought his advice and care, as previously feared procedures became possible. Microneurosurgery was estimated to have improved the outcome of neurosurgical operations by at least a factor of three [113]. However, this development did not come without resistance by established senior neurosurgeons. Decisive in the treatment of disturbances of cerebral circulation was the development of the extracranial-intracranial (EC-IC) bypass, which is described in more detail below. Furthermore, the microsurgical technique was combined with innovative approaches to the skull base and vascular tree; namely, the pterional approach for aneurysms of the anterior circulation [111] or basilar tip [110].

In 1971, Yaşargil was offered the chair of neurosurgery at the University of Berlin in Germany, but Krayenbühl insisted that he stay in Zürich, and he succeeded his teacher as chairman [106]. He asserted that he would endeavour to maintain the high standards set by Krayenbühl [105]. When he took over the position, he could count on a well-trained 180-person staff, some with decades of experience in neurosurgical patient care, as well as a solid average number of 70 admissions per week.

His work on the operating table, as well as his articles and books, were described as legendary by world leaders in the field. Alongside Harvey Cushing, Yaşargil was labelled “Man of the century”, as one of the two most important neurosurgeons of the twentieth century, for his immense contributions to modern microneurosurgery. His awards and honours have been listed elsewhere [104]. Without a doubt, he fundamentally influenced modern cerebral and medullary neurosurgery, which is based on methodologies developed at the USZ. Even today, many neurosurgeons all over the world use instruments developed in Zürich (e.g. Leyla bar, self-retaining retractor, mouthpiece for microscope position adjustment).

## USZ neurosurgery under the direction of Yasuhiro Yonekawa (1993–2007)

**Fig. 10:**
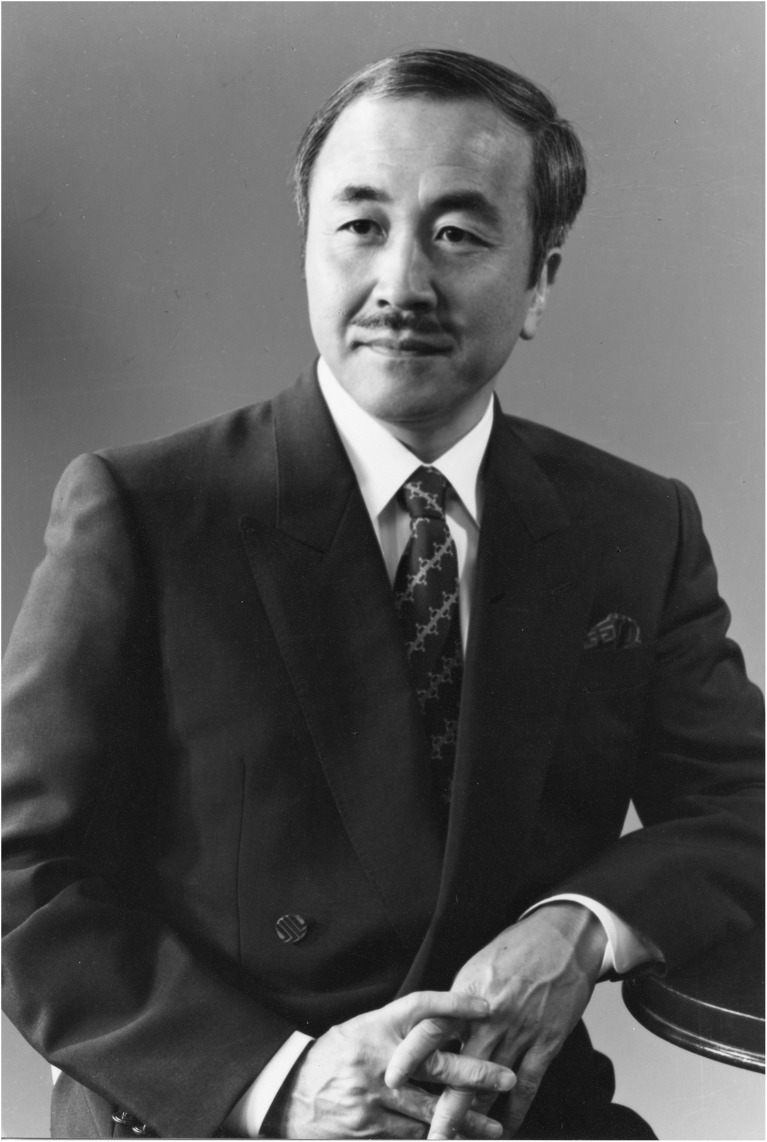
Portrait of Yasuhiro Yonekawa. B/w print. Photo credit: the family of Yasuhiro Yonekawa. Published with permission

Yasuhiro Yonekawa [born 12 November 1939 in Kyoto-fu (Japan); died 25 February 2017; Fig. 10] studied medicine in Japan. After receiving his medical and doctor’s degree in 1965, he was neurosurgical resident at the University Hospital of Kyoto (Japan) until 1969. He continued his neurosurgical training at USZ from 1970 onwards and was promoted attending in 1973. In this time, he was trained in microneurosurgery by M. Gazi Yaşargil until he returned to the University Hospital of Kyoto in 1977. He there wrote his habilitation on the topic, “Experimental intracranial transplantation of the omentum majus in dogs: a tentative new treatment for hydrocephalus and cerebral ischaemia”. In Kyoto, he was appointed associate professor for neurosurgery in 1981. In 1986, he was appointed Chair of the Department of Neurosurgery at the National Cardiovascular Centre in Osaka (Japan) and in January 1993 he took over the Department of Neurosurgery at USZ.

Based on the work of his predecessors, Yonekawa refined surgical treatment strategies for pathologies of the posterior part of the hippocampus [115], and the EC-IC bypass technique for moyamoya disease. He extended the pterional approach described by Yaşargil by adding extradural anterior clinoidectomy, which has become indispensable for the radical treatment of paraclinoid aneurysms [116].

He summarised the 70-year history of the department on the occasion of his retirement in 2007 [114]. The modern re-organisation of the department during his time led to a decrease in beds while maintaining the number of surgeries performed annually [114]. In his time, operating with the use of open magnetic resonance imaging (MRI) was introduced in Zürich, and congresses/workshops for intraoperative (io) imaging (held at the USZ in 2002; largely supported by René Bernays) and pituitary ademomas (held at the USZ in 1987; largely supported by Alex M. Landolt) were conducted, among others. He found great support by Hans-Georg Imhof in leading the department.

## USZ neurosurgery under the direction of Helmut Bertalanffy (2007–2010)

Helmut Bertalanffy had trained in Freiburg (Germany) and Tokyo (Japan) before he was Vice Chair of Neurosurgery in Aachen (Germany) and Chair of Neurosurgery in Marburg (Germany) from 1997 to 2007. Between 2007 and 2010, the USZ Department of Neurosurgery was headed by Helmut Bertalanffy, who is well known for his contributions to modern skull base surgery, and surgery on brain stem cavernomas in particular [12, 17].

## USZ neurosurgery under the ad interim direction of René Bernays (2010–2012)

Between 2010 and 2012, René Bernays led the department as interim chief. He is known for his contributions to the development of ioMRI, mainly in trans-sphenoidal pituitary surgery [6, 7].

## USZ neurosurgery under the direction of Luca Regli (since 2012)

Luca Regli trained in Lausanne. In Nicolas de Tribolet, he found not only a trainer but also a mentor. He later specialised in vascular microsurgery at the Mayo Clinic, Minnesota, with Thoralf M. Sundt (born 1930; died 1992) and Frederic B. Meyer. Visits led him to the neurovascular clinics of Robert F. Spetzler and Juha Hernesniemi. In 2008, at the retirement of Prof. C.A.F. Tulleken, he was appointed Head of Neurosurgery at the University Medical Centre of Utrecht, The Netherlands, one of the world’s leading academic neurovascular centres. In 2009, together with Karl Schaller, he held the European Lecture of the EANS on “promoting change in neurosurgery”. In October 2012, as the new Chair of the department, he brought change but also stability to USZ neurosurgery after a few turbulent years. His vast experience with minimally invasive and complex neurovascular surgery has particularly revitalised the neurovascular section at the USZ. Under his leadership, neurovascular surgery at the USZ has experienced a renaissance and the numbers of cerebrovascular procedures, particularly for the treatment of complex intracranial aneurysms, cerebrovascular bypasses [including excimer laser-assisted non-occlusive anastomosis (ELANA)] and arterio-venous malformations (AVMs), have again increased [96]. In addition, microsurgical carotid endarterectomy procedures, which he learned from the masters at the Mayo Clinic, have been added to the spectrum of USZ neurosurgical procedures. Luca Regli continues to develop microsurgical techniques for cerebrovascular indications. Under his leadership, the concept of “neurosurgery 3.0” moves the department into the next area, in which an armamentarium of sophisticated modern tools for intraoperative (io) imaging (high-definition io ultrasound, high-field 3-T ioMRI, io computed tomography [CT], three-dimensional endoscopy) as well as computer-assisted supports are ideally combined with established techniques (microsurgery, electrophysiology, etc.) to increase the precision and safety of complex neurosurgical procedures. Like Krayenbühl and Yaşargil [106, 108], Regli feels connected to and has a deep respect for the neurological specialty, perpetrating the family tradition of excellence in clinical neurosciences learned from his father, Franco Regli. Strong focus is placed on the complete and standardised assessment of patient outcome and complications for quality control [74]. This patient-centred philosophy and the on-going super-specialisation of neurosurgery within the “big fields” of cerebrovascular, neuro-oncological, functional, spine and paediatric surgery are key factors today to assert the leading position of a neurosurgical department in an increasingly competitive medico-economic environment.

## Discussion

Neurosurgeons have made continuous and notorious contributions to the care of patients suffering from cerebrovascular, neuro-oncological, functional and spinal disorders. Over recent decades, progress in endovascular and radiotherapeutical techniques have challenged the traditional role of microsurgery, pushing neurosurgeons to explore new avenues. In addition, an increasing number of healthcare providers competing for patients have led to a surplus of neurosurgical care in some regions, changing the referral patterns and postgraduate training models [8, 83]. Necessarily, transformations take place, but the spirit and the fundamental neurosurgical principles developed by our mentors continue to be key factors for success today and can be transferred to the present generation of neurosurgeons.

### Vascular neurosurgery

As described above, vascular neurosurgery has a special tradition and significance in Zurich [113]. Krayenbühl was among the first to perform cerebral angiographies and to demonstrate an aneurysm of the vertebro-basilar system in 1941 [35]. He describes in his thesis that even eminent specialists such as Dandy and Jefferson were sceptical about carotid ligation, the only available treatment in those days. Krayenbühl, however, considered the natural course of subarachnoid haemorrhage (SAH) as too disastrous, and promoted active treatment. He believed that

The extracranial ligation of (…) preferably the internal carotid artery for the care of intracranial aneurysms, remains the safest and surest method of attack for all the aneurysms of the sellar, parasellar and infraclinoid carotid tract, provided the efficiency of the collateral circulation is tested by prolonged sittings of compression of the common carotid in the neck. [47]

In his thesis, he states that until 1941, he had treated seven patients with saccular aneurysms and two patients with dural arterio-venous fistulas by carotid ligation. Only in rare instances did he choose intracranial approaches to directly puncture the sac of a sellar aneurysm, leading to aneurysm thrombosis (case 4 presented in his thesis), or to evacuate a giant middle cerebral artery (MCA) aneurysm by resection (case 11) [47].

Patient numbers grew. From his operative records, we know that up until 1945 he had treated 77 patients with intracranial aneurysms, most of them with carotid ligation [33]. He mentions the report of Dandy’s clipping [23], but did not use this technique himself at that time. In 1965, Krayenbühl and Yaşargil [49] summarised their broad experience gained after the early introduction of cerebral angiography at the USZ in their textbook *Die zerebrale Angiographie*. Worldwide, this opus was the standard reference textbook for many years [113]. Both were strong proponents of neuroradiology as a new, distinct specialty within the clinical neurosciences, and together with Anton Valavanis—a pioneer of neuroradiology and endovascular interventions—built a mutually stimulating environment at USZ [92].

Krayenbühl was among the first to realise the great potential of microsurgical techniques in neurosurgery. He sent a young associate, Yaşargil, to the USA to gain expertise in this field (see above). Insight into his thoughts can be found in the record of his speech entitled “The place of microsurgical technique in neurological surgery”, given on the occasion of the Fifth Sir Hugh Cairns Memorial Lecture, on 30 May 1969 in London [48]. He stressed that the use of the microscope in neurosurgery is “an extremely useful adjunct to surgery”, and that especially the good illumination, unobstructed vision, and improved view of the surgical site are “of inestimable value to the surgeon” [48]. Krayenbühl mentions that in order to apply microsurgery to the field of vascular neurosurgery, in which he sees “the type of indication *par excellence*”, instruments and micro-suture material must still be developed further. As disadvantages in the use of the operating microscope, due to which he also understands the reluctance of many to use the instrument more widely, he lists the following:

1. The surgeon’s head and body must work often for prolonged periods of time in a position to which he is not previously accustomed.

2. Due to the angle of the lens, a new technique of coordination of the surgeon’s hands and eyes must be learned.

3. The use of precise micro-instruments must be mastered.

4. Work is done in a small operative field.

5. Present microscopes are bulky and difficult to handle.

6, Time and patience are necessary, and it is important to work in a quiet, disciplined environment.

7, Caution is particularly required to avoid contamination of the operative field. [48]

In his presentation, Krayenbühl discusses practical aspects of the application of microsurgical techniques that go beyond the constraints of this article. However, he cites Cairns by saying that “Surgery is and must be always an art, but its progress and thus its vitality depend on the maximum application to it of the methods and discoveries of science”. A statement that remains valid today.

The EC-IC bypass, comprising the anastomosis of the superior temporal artery (STA) and MCA, was technically developed by Yaşargil and Donaghy. After extensive laboratory practice, Yaşargil performed the first STA-MCA bypass on 30 October 1967, in Zürich, on a patient with Marfan’s syndrome and complete occlusion of the MCA [64]. The first case of moyamoya disease treated with a direct STA-MCA bypass was also performed by Yaşargil in 1972, on a 4-year-old child who showed remarkable improvement following the procedure [64]. After the International Cooperative Study (1985) failed to prove the efficacy of this method in preventing ischaemic stroke for artherosclerotic disease [89], it nonetheless retained an important place in the treatment of highly selected patients with compromised cerebrovascular reserve capacity [24]. The hospital thus benefited from a national and international reputation as a referral centre for complex aneurysms, AVMs, atherosclerotic occlusions and moyamoya disease. Revascularisation techniques were developed for the anterior cerebral artery, the posterior cerebral artery and posterior circulation [113], resulting in a stable number of referrals and increased expertise over time [113]. As successor of Tulleken in Utrecht, Regli had the unique opportunity to participate in the further refinements of non-occlusive revascularisation techniques and introduced in 2012 the ELANA bypass technique at the USZ [13, 95–97].

Due to the rise of effective endovascular treatment techniques, the decreasing number of microsurgical occlusions of intracranial aneurysms was also observed at the USZ. The number of aneurysm clippings decreased from 100 (1993) to 60 (2006) per year, while the number of aneurysms treated by coiling rose from 40 to 60 in the same time period [113]. The same applied to the treatment of AVMs. Due to Anton Valavanis’ particular expertise in super-selective embolisation [93, 94], the number of endovascularly treated patients with AVMs rose steadily and reached 70 per year, with fewer patients treated surgically (20 per year) at the end of the last decade. The role of cerebrovascular microsurgery is being redefined constantly, by analysing subgroups of patients who benefit from the microsurgical treatment of intracranial aneurysms, AVMs and from cerebral revascularisation [24]. Cerebrovascular referral centres will see more and more patients with complex lesions.

### Epilepsy neurosurgery

Krayenbühl was interested in epilepsy surgery and laid the foundation for a further focus at the USZ in the 1940s–1950s. During the first 18 years of the department, a total of 2,336 patients with epilepsy were assessed and treated. He promoted complete anterior temporal lobectomies, keeping the vein of Labbé as a posterior boundary [101]. During most of the operations, corticography was applied in the way Wilder Penfield performed his operations, while Krayenbühl limited the cortical exposure whenever possible [25]. The neurophysiological data were interpreted by the neurophysiologist Rudolph M. Hess (son of the Nobel prize winner Walter Rudolf Hess), who closely collaborated with the neurosurgical department [75]. Krayenbühl’s surgical decisions were always based on the convergence of clinical features and electroencephalography (EEG) findings [25].

At the beginning of the 1970s, stereotactic electroencephalography (SEEG) was frequently used during surgery to support lesionectomies and more extended anterior temporal lobectomies. The experience gained with this method, in combination with the introduction of the operative microscope and the new spectrum of micro-instruments available, led to the development of more tailored methods from 1967 onwards. Thus, the selective trans-sylvian amygdalohippocampectomy (SAHE) for medically refractory epilepsy was proposed by Yaşargil, based on the idea that the entorhinal cortex is a main area of seizure onset [103, 112]. In 1973, the first SAHE was performed at the USZ [75]. It was shown that SAHE, with a preoperative Wada test for speech and memory function, as well as intra-operative EEG recordings, resulted in satisfactory results for seizure outcomes, and in better cognitive function preservation [32]. Nowadays, SAHE is a standard operative procedure in temporal lobe epilepsy, and is used all over the world. For pathologies of the posterior hippocampus, the supra-cerebellar transtentorial approach was developed by Yonekawa and co-workers [115], allowing sufficient access without damage to the antero-lateral temporal structures. The encouraging results obtained with SAHE stimulated and inspired the later development and introduction of the foramen ovale (FO) electrode (in 1983), a semi-invasive method for the preoperative assessment of limbic epilepsy. Up until 1998, FO electrodes were used in 214 patients at the USZ, and the technique became popular worldwide [25, 102]. Due to advances in neuroimaging, the use of the FO electrode has again declined over recent decades [42].

In 1989, the 50-year expertise of the USZ in vascular anatomy and angiography converged in a remarkable way in the study and surgical treatment of epilepsy. The introduction of the selective and superselective amytal memory test at the USZ (Anton Valavanis) allowed for more precise presurgical language and memory investigations (the Wada test). Detailed pre-surgical workup with the Wada test, MRI (standard since 1983), as well as functional imaging by single-photon emission computerised tomography (SPECT) and positron emission tomography (PET; available since 1988) led to an improved selection of surgical candidates [25, 31].

### Skull base neurosurgery

For skull base surgery, the USZ benefited from the exceptional technical skills of both Yaşargil and Ugo Fisch (head of ENT at the USZ from 1970 to 1999), who took care of pathologies both inside and outside the dura, respectively, but also operated together in complex cases [106]. For the trans-sphenoidal surgical treatment of the pituitary, Krayenbühl, Yaşargil and Fisch mutually exchanged their experience with Jules Hardy from Montreal. Alex Landolt continued trans-sphenoidal surgery at the USZ from 1971 onwards, gathering experience in the field by operating on more than 2,000 patients. He was an internationally renowned expert for trans-sphenoidal surgery, who revised the nomenclature of pituitary tumours in 1975 [52, 100]. Yaşargil himself always expressed his deep respect for skull base surgery, especially in the region of the cavernous sinus, and characterised his own surgical skills as moderate for these anatomical regions. It was not until modern high-speed pencil-like drills came on the scene that Osama Al-Mefty, Madjid Samii, Vinko Dolenc, and other collaborators and visitors to the USZ could develop specific skull-base approaches and push the limits for the good of their patients [106]. Concerning tumours of the cranial nerves, the proof of efficacy of radiosurgery has changed the game for the management (in particular for vestibular schwannomas). Nowadays, small and usually well-operable cases are primarily irradiated, and the larger lesions are referred for surgical treatment. Concerning neoplastic lesions of the skull base (meningiomas in particular), surgery can be performed more safely in patients with a high risk for surgical morbidity [26], as tumour remnants can be controlled by conformational radiation therapy. In skull-base surgery, modern micro-neurosurgical concepts following the “maximal safe resection” strategy differ from the treatment strategies of our predecessors, which aimed at radical tumour removal and would face the risk of significant morbidity.

### Neuro-oncological care

In his operative records, Krayenbühl noted the extirpation of 265 tumours up until 1945, of which 88 were labelled complete, 87 were partial and 8 were “intracapsular” resections (remaining unknown) [33]. In 1937, he introduced the smear technique, enabling a prompt histological diagnosis during surgical exploration. During neuro-oncological procedures, he immediately sent the specimens obtained to the neuropathology (and to his own) laboratory for a first diagnosis [106]. His operative records prove that palliative cranial decompressions for inoperable tumours—as promoted by both Horsley [40] and Cushing [21, 29]—were not uncommon for treating patients with signs of elevated ICP [33].

Yaşargil outlined his theories on some of the disease patterns of CNS neoplasms in his textbooks *Microneurosurgery vols. IVA and IVB*, which were studied by generations of clinicians and researchers [107, 108]. Surgical care was improved thanks to microsurgical techniques and intraoperative neurophysiological monitoring. Important steps forward were made using new techniques for low- and high-definition io-imaging and neuronavigation. The USZ obtained the first General Electric open ioMRI apparatus in Europe (and second in the world after Brigham Hospital in Boston, USA). Collaborators of the USZ succeeded in developing ioMRI-compatible instruments to transfer the ioMRI technique from the laboratory to the clinical setting [9], and intraoperative ultrasound (ioUS) and ioMRI now allow for the intraprocedural evaluation of extent of resection [63, 77, 78]. Awake surgery for brain tumours in eloquent areas is used as needed. The department benefits from clinical and experimental collaborative research with USZ oncologists and neurologists specialised in the neuro-oncological field, such as Roger Stupp and Michael Weller, who have contributed considerably to the evidence existing today for adjuvant therapy, leading to the improved overall survival of patients with brain tumours [34, 87]. Interdisciplinary collaboration, shared expertise through dedicated tumour boards, and patient care in neuro-oncology has clearly evolved over recent decades.

### Spinal care

Despite being commonly perceived as a centre specialising in cranial procedures, the department of neurosurgery at the USZ has a long-standing tradition in the surgical care of spinal pathologies. USZ neurologist Otto Veraguth played an important role in one of the first surgeries ever performed for a herniated lumbar disc (see above) [38, 86]. Moreover, the method of laminectomy was introduced at the USZ on 31 January 1938, to remove a spinal meningioma. From then on, Krayenbühl frequently used laminectomies to treat spinal tumours, disc herniations, and inflammatory diseases [33]. As already pointed out by Yonekawa, about 250 lumbar disc surgeries were performed each year in Krayenbühl’s era [113], as well as more complex procedures such as anterolateral chordotomies for the palliative treatment of intolerable spinal pain (from 1942 onwards) [33]. The department offers the whole spectrum of spinal surgical procedures, including procedures on the degenerative spine, but also complex spinal neuro-oncological procedures. In 2015, the installation of an ioCT pursues the department’s strategy of high-definition intraoperative imaging, and offers increased precision for image-guided spinal stabilisation.

### Functional surgery

Functional neurosurgery has been an USZ “hot topic” for a long time. In three publications between 1884 and 1895, Krönlein described surgical techniques to resect the trigeminal nerve [36]. Krayenbühl performed cranial nerve *exérèse* (= nerve extraction) 17 times between 1941 and 1945, for supraorbital, trigeminal and occipital neuralgia, as well as for *neuralgia paraesthetica*. He also tried a trigeminal tractotomy on 5 February 1942, as described by Sjöqvist [81], by suboccipital craniotomy and incision of the lateral medulla oblongata, next to the cerebellar tonsil [33]. For a long time, Krayenbühl’s preferred method, however, was the transection of the trigeminal nerve as described by Spiller and Frazier [82], which he performed 131 times until 1941 [33]. This was not without complications, and triggering the trigeminal-cardiac reflex, with the resulting hypotension, vasoconstriction, and apoplexy, was a common reason for surgical morbidity and mortality in his series [1]. The so-called “Dandy’s surgery” [73] for transection of the acoustic nerve in Manière’s disease via retrosigmoid craniotomy was first conducted at the USZ on 21 November 1941 [33]. In addition, posterior rhizotomy for the treatment of intercostal neuralgia, sympathectomy for the treatment of complex regional pain syndrome, and “Stoffel’s surgery” for the transection of peripheral nerves to treat spastic paresis are described from 1940 [33]. In the 1940s, the treatment of hyperkinetic disorders was still in its infancy. Horsley was among the few that attempted surgical treatment for these indications. In 1909, he resected the precentral gyrus in a 15-year-old boy with hemiathetosis, with a good clinical result [98]. Krayenbühl’s operative records indicate that he performed “Horsley’s surgery” only once, on 21 January 1938, for hemiathetosis [33].

During the founding years of the department, lobectomy for the treatment of psychiatric disorders was popularised in Europe by the Swiss psychiatrist Gottlieb Burckhardt and the Portuguese neurologist Egaz Moniz (Fig. 7) [10]. According to his operative records, Krayenbühl did not operate on patients for psychiatric indications. He did, however, gain first-hand experience in stereotactic surgery in the regions of the thalamus and globus pallidus, as evident from ten articles published between 1960 and 1966 on the results of stereotactic surgery in patients with Parkinson’s disease [80]. Under Yaşargil, who himself learned it from Traugott Riechert and his team in Freiburg (Germany), this rising branch of functional neurosurgery further developed with the addition of intraoperative electrophysiology. Jean Siegfried (1931–2014) should be mentioned here, known for his innovative and pioneering work in the field of deep brain stimulation (DBS) for chronic pain and movement disorders, as well as in stereotactic and functional neurosurgery [11]. His professional contributions were awarded with several prizes, among them the prestigious Otfrid Foerster Medal in 2003. Moreover, Daniel Jeanmonod was an instigator of functional neurosurgery at the USZ from 1989 to 2009, where his studies on the electrophysiological determination of target points gained resonance on an international level [41]. As part of USZ neurosurgery, he helped to develop transcranial magnetic resonance (MR)-guided high-intensity focused ultrasound (tcMRgHIFU) as a novel, non-invasive treatment strategy for thermal ablation in various brain diseases [58]. Today, functional neurosurgery and DBS continue to be regularly performed at the USZ.

### Hydrocephalus and CSF disorders

The USZ Neurosurgery Department treats over 100 patients with cerebro-spinal fluid (CSF) disorders per year [113]. Given the distribution of indications, ventriculo-peritoneal shunting as popularised by Cushing remains the most frequent procedure for permanent CSF diversion (Cushing performed drainage into the retroperitoneal space [21]). Krayenbühl used techniques to avoid direct cerebral access whenever feasible (communicating hydrocephalus; Fig. 6d). Third ventriculostomy for the treatment of occlusive triventricular hydrocephalus was first described in 1920 by Dandy [22]. This treatment was based on simple and fundamental physiological and surgical principles. It avoided the use and permanent implantation of foreign material within the CNS and other body cavities. Therefore, Krayenbühl soon adapted this technique for use in patients with hydrocephalus due to large midbrain tumours (4 October 1938) and Chiari malformation (27 October 1939) [33].

### Neuro-intensive care medicine

Increasingly complex neurosurgical procedures and the growing understanding of cerebral physiopathology, together with a shift towards better neuro-resuscitation and more aggressive treatment of patients with high-grade SAH called for a specialised neuro-intensive care unit (neuro-ICU) with dedicated specialists. For nearly 20 years, Emanuela Keller and her neuro-ICU team—as part of the neurosurgical department—have made substantial contributions to the success of the department. Emanuela Keller has conducted and contributed to research on multimodal cerebral monitoring (including data mining and cognitive computing), and the optimisation of neuroprotective therapies and inflammatory responses after SAH [61, 79].

### Postgraduate training

Microsurgical techniques and selective approaches to the cerebral vascular tree have been taught in numerous hands-on courses offered at the USZ, in collaboration with the Anatomical Institute of the University of Zurich. This includes the legendary “Microsurgery Training Course” initiated by Yaşargil, which has taken place in Zurich since 1968 with support of Ms. Rosmarie Frick, Hans-Georg Imhof, Yasuhiro Yonekawa and others. The course was attended by more than 3,000 participants until Yaşargil’s retirement in 1992. Under Luca Regli’s guidance, the course continues to attract neurosurgeons and other specialists from around the world. Moreover, courses on white matter dissection (Niklaus Krayenbühl) and microsurgical approaches to the skull base and the brainstem (Oliver Bozinov) aim to improve the understanding of precise topographical microanatomy and to master microsurgical approaches continuing to convey the microsurgical skills of the Zurich school.

### Surgical illustration

Since the foundation of USZ neurosurgery, medical illustrators have captured innumerable procedures, augmenting undergraduate and postgraduate education and enriching the medical publications of members of the department (Figs. 4 and 6). Werner Bärtschi (Fig. 4) was followed by Hans Peter Weber (1914–2012; Fig. 6), who worked as a medical illustrator with both Krayenbühl and Yaşargil from 1947 to 1979. Peter Roth has been the medical illustrator for the department since 1974 [106]. The educational value of his illustrations in books and journals are well recognised by the neurosurgical community.

## The founding of the USZ in the context of European neurosurgery

The neurosurgical department at the USZ was among the first of its kind in Europe [2], and was founded along the lines of the independent departments established in London, England, in 1886 (Victor Horsley) [70], Berlin, Germany, in 1900 (Fedor Krause), St.Petersburg, Russia, in 1910 (Pussel) [54], Breslau, former Germany, in 1911 (today’s Wrocław, Poland; Otfrid Foerster; Fig. 7) [19] and Istanbul, Turkey, in 1923 (Abdulkadir Cahit Tuner) [62]. Ludvig Puusepp (Fig. 7) was a Professor of Neurology at the University of Tartu with a strong interest in surgery, who performed the first brain tumour operation in Estonia on 30 April 1921, for a right-sided cerebello-pontine angle mass. The Tartu clinic remained the key Baltic centre for neurosurgery until 1940, but the exact time-point for the founding of a dedicated neurosurgery department in Estonia remains unknown to the authors [91]. Other early departments were established in 1930 in Dublin, Ireland (Adams Andrew McConnell) [53], and in 1933 in Socola, Romania (Moruzzi) [71]. In France, Antony Chipault (1866–1920) was destined to become the father of French neurosurgery for his important clinical and scientific contributions to the field, but he retired early due to an unknown neurological disease [4]. It was thus the general surgeons Thierry de Martel (1876–1940; Fig. 7) and Clovis Vincent (1879–1947; Fig. 7) who paved the way for neurosurgery to become a specialty in France. A specialised department at *L’hôpital de la Pitié*, in Paris was created in 1933 and was headed by Vincent; a university chair in neurosurgery followed in 1938 [15]. In Germany, Wilhelm Tönnis founded the department of neurosurgery in Würzburg in 1934 [19], and in 1935, departments in Stockholm, Sweden (Herbert Olivecrona), and Bucharest, Romania (Dimitrie Bagdasar) [60], followed, as well as a department in Frankfurt, Germany in 1936 (Traugott Riechert) [19]. In the Netherlands, early representatives of neurosurgery were the two US-trained physicians, Ignaz Oljenick [(1888–1981; started neurosurgical activity in Amsterdam in Bernard Brouwer’s (1881–1949) neurological department in 1929)], Ferdinand Verbeek (1902–1958; started neurosurgical activity in Groningen in 1935), as well as Cornelis Hendrikus Lenshoek (1902–1969), who established the French school of neurosurgery in Utrecht in 1936 [30, 43]. The German invasion during the Second World War disturbed the development of neurosurgery in The Netherlands considerably. Departments founded after 1937 include those in Oxford, England (Hugh Cairns, 1938) [69], Bonn, Germany (Peter Röttgen, 1938) [19], Belgrade, Serbia (Milivoje Kostić, 1938) [71], Athens, Greece (Heliades K., 1939) [71], Kolozsvar, Romania (1941) [20], Sofia, Bulgaria (Philip Philipov, 1942) [71] and Zagreb, Croatia (Danko Riessner, 1945) [71], among others. Italian neurosurgery has a long tradition, as recently reported in a comprehensive review [59], with contemporary neurosurgery evolving in parallel to the development of clinical neurology, neuropathology, neurophysiology and neuro-anaesthesiology. The most important schools were reported to be located in Florence, Bologna, Pisa, Turin and Padua–Venice; later also in Pavia, Verona, Milan, Rome and Naples [59], whereas the exact date of foundation remains unknown. In Spain, Barcia Goyannes began a neurosurgical practice in Valencia in 1957. His efforts, and those of Tolosa and A. Ley (Barcelona; Fig. 7) and Obrador and E. Ley (Madrid), led to the establishment of the first dedicated neurosurgery units on the Iberian Peninsula [117]. In Belgium, the first neurosurgical department was built by Paul Martin (1891–1968; Fig. 8) at the Institut Héger-Bordet (Université Libre de Bruxelles) in 1948, following his training in Halsted’s and Cushing’s clinics [14]. This list is not exhaustive, but it serves to demonstrate that Krayenbühl knew many of those early founders of European neurosurgery, as likewise documented by Figs. 7 and 8. They mutually influenced each other, often maintained professional and personal relationships, and built a common ground to promote modern neurosurgery in Europe.

### Why establish neurosurgery as a specialty in Switzerland?

What were Krayenbühl’s motivations for the ambitious goal of founding neurosurgery as distinct specialty in Switzerland? The social and political situation was unfavourable: the 1930s were characterised by high unemployment (1936: 124,000 unemployed) and a decline in the birth rate led demographers to predict a significant drop in the Swiss population. Everyone economised as the Swiss Franc lost 30% of its value. There was a possibility that Switzerland would get involved in the war: Hitler had violated the Treaty of Versailles and re-militarised the Rhineland. Germany was armed and the future looked dismal.

We find detailed information on this question in the article by Gerhard Weber, Krayenbühl’s former associate and the first chair of the neurosurgical department in St. Gallen [99]. Paul Clairmont realised that globally, neurosurgery was on the rise and that his surgical clinic could not meet demand in terms of diagnostic tools and volume. He provided the necessary political support for this endeavour. In the USZ report of 1936, he states:

*Die Neurochirurgie hat sich in den letzten zwei Jahrzehnten vor allem durch die Arbeiten des amerikanischen Chirurgen Cushing zu einem Spezialgebiet der Chirurgie entwickelt. Der Neurochirurg muss einerseits im Stande sein, aufgrund seiner neurologischen Kenntnisse die Diagnose der Krankheiten des Gehirns und des Rückenmarks selbst zu stellen und als Chirurg die Indikation zur Operation zu beurteilen, und andererseits mit einer ganz besonderen operativen Technik vertraut sein, die sich in den letzten Jahren herausgebildet hat. So sind vorerst in den angelsächsischen Ländern, in jüngster Zeit aber auch in Schweden, Holland, Frankreich und Deutschland zahlreiche neurochirurgische Kliniken gegründet worden. (Cited after* [99]*)*

(Neurosurgery has developed as special field of surgery over the last two decades, mainly through the work of the American surgeon, Cushing. The neurosurgeon must, on the one hand, be able to diagnose the diseases of the brain and the spinal cord by his neurological knowledge and assess—as a surgeon—the indication for an operation, but on the other hand he must be familiar with a very special operative technique that has evolved over recent years. For the time being, numerous neurosurgical clinics have been established in the Anglo-Saxon countries, but also in Sweden, Holland, France and Germany.)

The same report gives evidence of an early example of the ambitious idea for the centralisation of highly specialised care in Switzerland. The text is believed to originate from Krayenbühl himself:

*Durch die Errichtung einer neurochirurgischen Station im Hegibach soll die Zentralisation chirurgischer Fälle unseres Landes in einer besonders eingerichteten Klinik ermöglicht und die Kranken dank einer guten Organisation und besonderen technischen Ausrüstung einer best-möglichen Behandlung zugeführt werden*. (Cited after [99])

(The establishment of a neurosurgical station in the Hegibach building is intended to enable the centralisation of the surgical cases of our country in a specially established clinic, and to provide patients with the best possible treatment thanks to good organisation and special technical equipment.)

Krayenbühl, well-trained in pathology, psychiatry and neurology was the ideal candidate. He was unsatisfied with the “therapeutic nihilism” of neurology, where the highest ambitions at that time were to confirm detailed diagnostic assessment by autopsy. He felt the urge to apply active neurosurgical treatment methods, but tested his manual dexterity in general surgery before he took on the neurosurgical fellowship with Cairns (see above). He was initially employed as *Volontärarzt* (not receiving any salary), but helped to finance the expenses of the new clinic. Him and his wife would design the patients’ rooms, including curtains and pictures. Besides indicating his generosity, these facts remind us of his maxim “*Soignez les détails*” (“Cherish the details”).

He began his task in Zurich as a one-man show. He practiced holistic medicine, examining his patients from an internal medicine, psychiatric, neurological, ophthalmological and radiological standpoint. He performed the necessary diagnostic tests himself. He would discuss the results and therapeutic options with the patient and their relatives, aiming for informed consent. For the operation, he would frequently invite the involved family physicians to attend. These often accepted the offer, as these kinds of procedures were unknown to most in Switzerland at that time. He would also personally supervise the postoperative care of all his patients. At 7 a.m. he would appear for morning rounds, and shortly before 11 p.m. he would go on evening rounds. He trained his OR staff himself, especially his scrub nurses who would intubate, administer anaesthesia, and examine blood and CSF samples. He instructed the nurses in the ward in important aspects of neurosurgical care, as dealing with patients with motor deficits or reduced vigilance was uncommon. In contrast to many surgeons of his time, Krayenbühl regarded decubitus as a sign of bad patient care and did not accept it in his clinic. He promoted neurological rehabilitation and the establishment of intensive care units.

It goes without saying that this work could only be accomplished by intense weekly working hours. He regarded neurosurgery not as a job to achieve financial success, but as a vocation, as an inner calling of complete devotion to his patients.

### Lessons learned from reflecting on the past

Reflecting on the founders and promoters of neurosurgery in Switzerland, we realise that the common denominators of success are the same in the past as they are today: dedication to patients and neuroscience, interdisciplinary work, exchanges between neurosurgical centres and colleagues, visits (today observerships) and travels (today fellowships) to see and learn new and different ways, a pioneering spirit, and both the development and introduction of new techniques into clinical work (Table 2). Remaining local, wearing blinders to innovation and refraining from implementing innovation has not been rewarding in the past. Fortunately, this type of attitude is less likely for the present generation of neurosurgeons, considering also the ample opportunities to build up and foster professional networks, e.g. during events organised by the EANS, AANS, CNS and other professional organisations.

Despite the intriguing similarities of the past and today, some fundamental changes are taking place in modern health systems and therefore also in modern neurosurgery. Patients are much better informed about their disease and treatment options, leading to a distinct change of the physician-patient relationship. Today’s patients have evolved into informed customers and service partners focused on patient-relevant results. Health systems are increasingly driven by competition based on results and cost efficiency, similar to market dynamics. Patients no longer only expect the performance of a surgical procedure, but the delivery of results topped with an excellent service. Quality of care has become increasingly scrutinised, and treatment results that are directly relevant to the patient are targeted. The complete removal of a brain tumour in a risky location or a perfectly decompressed spinal canal with correctly placed fusion material is of no benefit to the patient if his/her functional impairment and quality of life is not significantly improved by the procedure. This development is reflected in the increasing number of publications in the modern neurosurgical literature that employ patient-rated or objective outcome measures over surgeon-rated treatment success [5, 28, 84]. In this context, there is a continuing need for innovation in techniques and performance, supporting the designation of supra-regional centres for highly specialised care.

## Conclusions

The founding of neurosurgery at the USZ allowed the Swiss school of neurosurgery to develop and influenced the rise of modern neurosurgery in Europe. Over the decades, it has laid the grounds for important developments in the specialty; in particular, in the areas of vascular, skull base, epilepsy and neuro-oncological surgery. As such, it continues to be among the top teaching institutions not only in Switzerland, but also in Europe, and is visited by many students, postdocs and surgical fellows. Looking back, one realises that to go forward, neurosurgery today needs to integrate both the characteristics of success of yesterday and modern visionary concepts.

## Electronic supplementary material

Supplementary Figure 1 Group photo of Hugo Krayenbühl’s primary school class. The 8-year-old Hugo Krayenbühl is second from the left, in the last row. The picture is taken in Zihlschlacht (Switzerland), 1910. Black/white print on carton. Photographer unknown. From [33]. (JPEG 470 KB)

Supplementary Figure 2 1918 confirmands with pastor Pfisterer. The 16-year-old Hugo Krayenbühl is fifth from the right, in the second last row. The picture was taken in Bischofszell (Switzerland), in front of the “Stiftskirche St. Pelagius”, 1918. Black/white print on carton. Photographer unknown. Photo credit: Archiv für Medizingeschichte Universität Zürich (AfMZH) PN 83.02. Published with permission. (GIF 26.1 MB)

High resolution image (TIFF 43.4 MB)

Supplementary Figure 3 Hugo Krayenbühl and his teacher Hugh Cairns. Photo taken in the Swiss Alps. Exact time and location unknown. From [33]. (JPEG 424 KB)

Supplementary Figure 4 Scan of the front page of Hugo Krayenbühl’s 1941 habilitation on cerebral aneurysms. (JPEG 390 KB)

Supplementary Figure 5 Photo of the front page of Hugo Krayenbühl’s “Operationsbuch” containing his hand-written surgical records. From [33]. (JPEG 210 KB)

Supplementary Figure 6 Illustration of the rise in number of diagnostic (**A**) and therapeutic interventions (**B**) from the founding year of 1937 until 1945. The source of this data is the hand-written notes of Krayenbühl’s “*Operationsbuch*” (=surgical records) (adapted from [33]). “Spiller-Frazier” refers to the transection of the trigeminal nerve as described by Spiller and Frazier. [82] (JPEG 137 KB)

High resolution image (EPS 88.8 KB)

(JPEG 196 KB)

High resolution image (EPS 110 KB)

Supplementary Figure 7 Hugo Krayenbühl after his last surgery. Photo taken in 1973. From [33]. (JPEG 380 KB)
