# Supplementary figures and images for: UniversitätsSpital Zürich: 80 years of neurosurgical patient care in Switzerland
Source: Acta Neurochir (Wien). 2017 Nov 13;160(1):3–22. doi: 10.1007/s00701-017-3357-z (PMC5735218; doi:10.1007/s00701-017-3357-z)

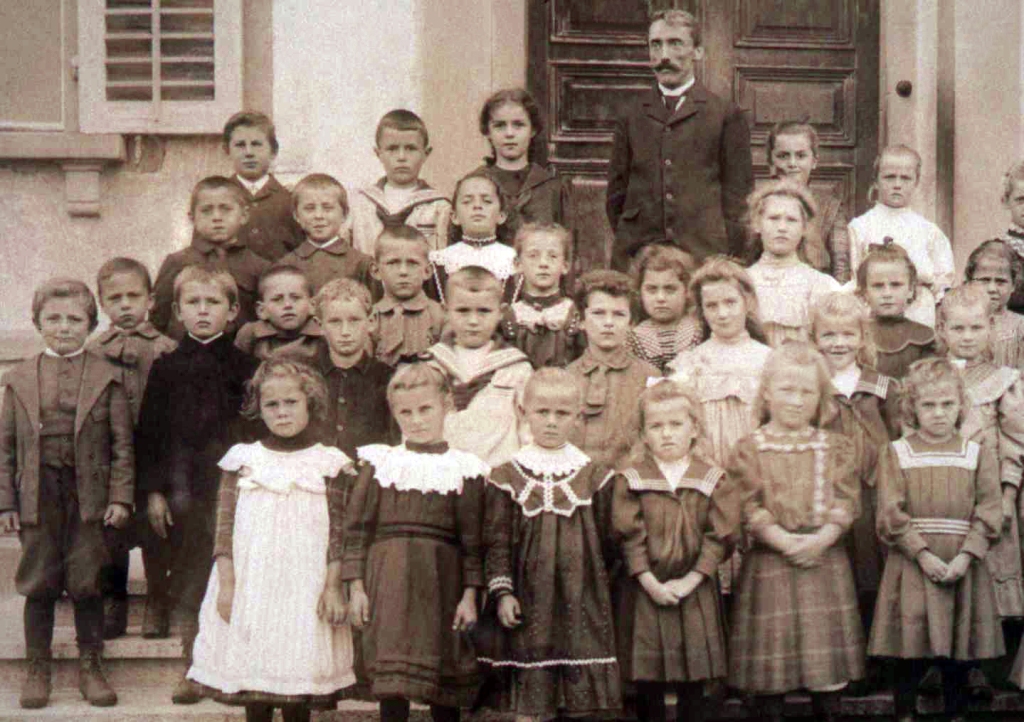

Supplement: Supplementary file 1 — Group photo of Hugo Krayenbühl’s primary school class. The 8-year-old Hugo Krayenbühl is second from the left, in the last row. The picture is taken in Zihlschlacht (Switzerland), 1910. Black/white print on carton. Photographer unknown. From [33]. (JPEG 470 KB) [file 701_2017_3357_MOESM1_ESM.jpg]

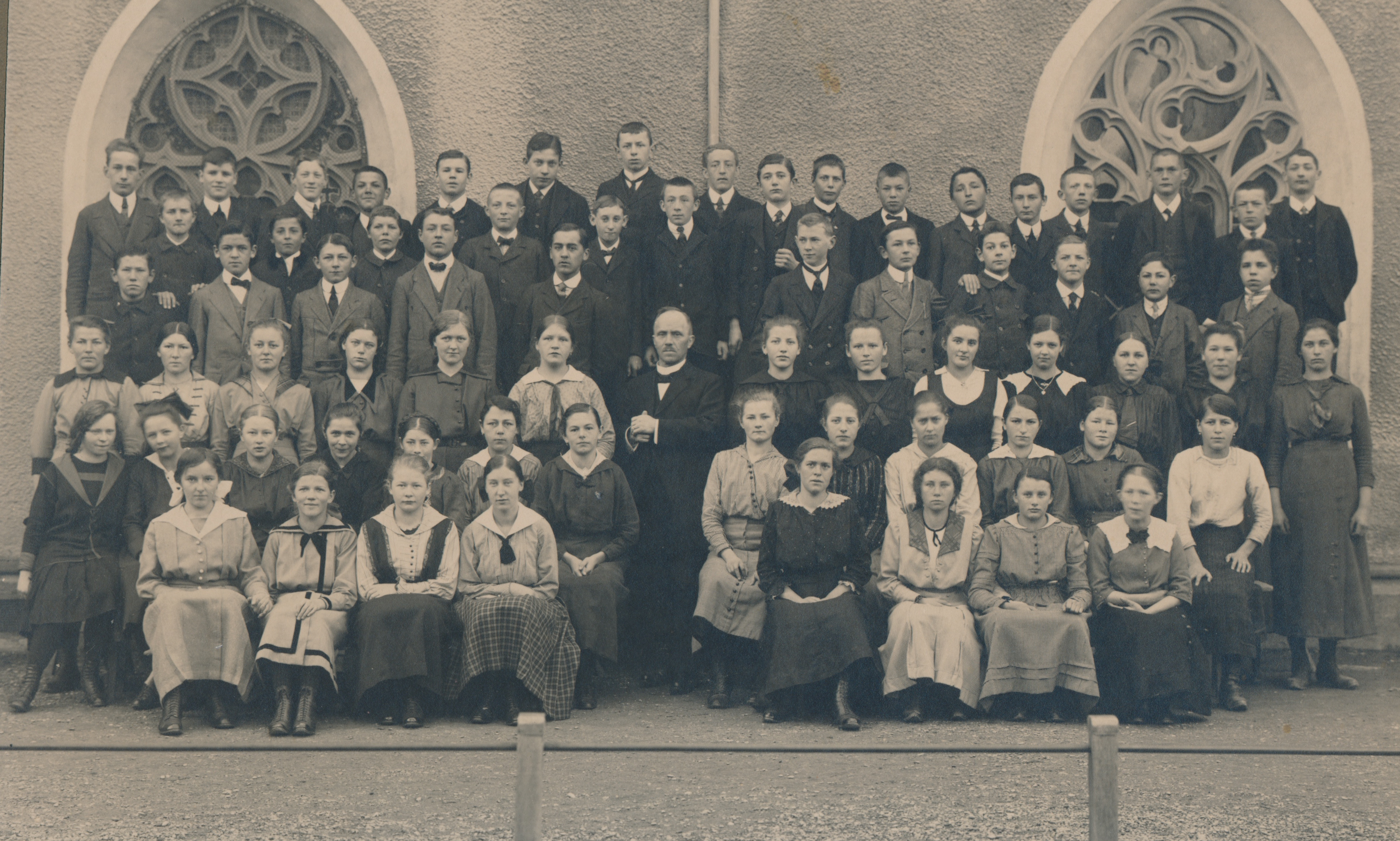

Supplement: Supplementary file 2 — 1918 confirmands with pastor Pfisterer. The 16-year-old Hugo Krayenbühl is fifth from the right, in the second last row. The picture was taken in Bischofszell (Switzerland), in front of the “Stiftskirche St. Pelagius”, 1918. Black/white print on carton. Photographer unknown. Photo credit: Archiv für Medizingeschichte Universität Zürich (AfMZH) PN 83.02. Published with permission. (GIF 26.1 MB) [file 701_2017_3357_Fig11_ESM.gif]

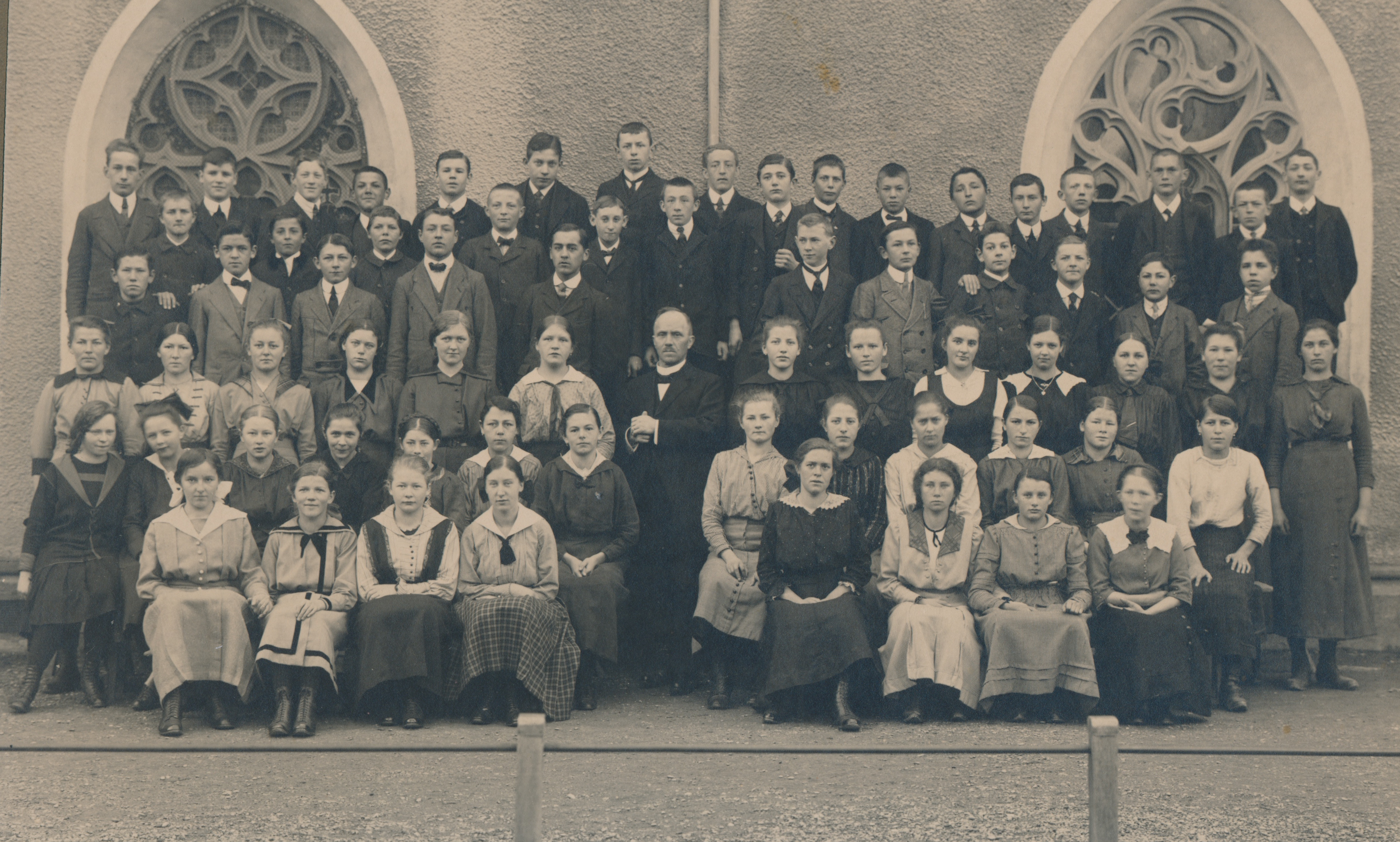

Supplement: Supplementary file 3 — High resolution image (TIFF 43.4 MB) [file 701_2017_3357_MOESM2_ESM.tiff]

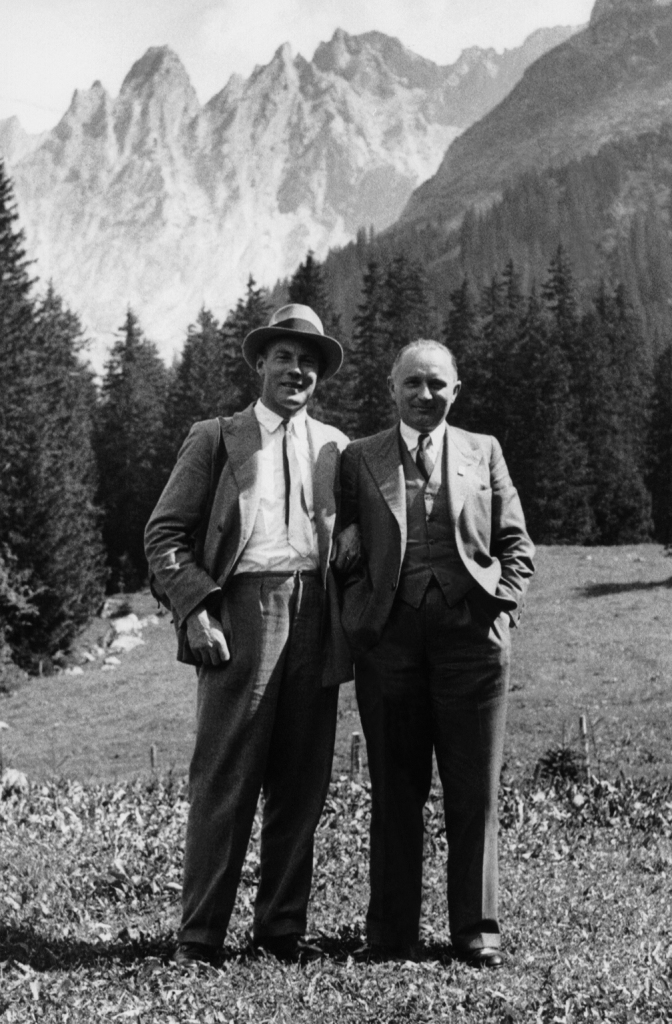

Supplement: Supplementary file 4 — Hugo Krayenbühl and his teacher Hugh Cairns. Photo taken in the Swiss Alps. Exact time and location unknown. From [33]. (JPEG 424 KB) [file 701_2017_3357_MOESM3_ESM.jpg]

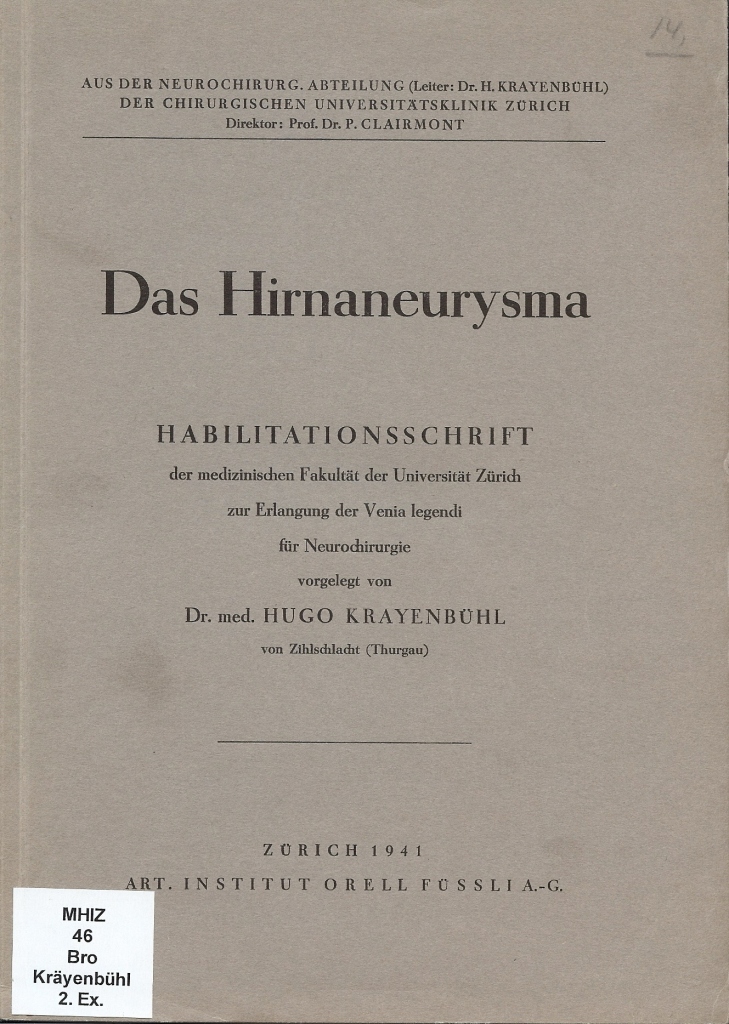

Supplement: Supplementary file 5 — Scan of the front page of Hugo Krayenbühl’s 1941 habilitation on cerebral aneurysms. (JPEG 390 KB) [file 701_2017_3357_MOESM4_ESM.jpg]

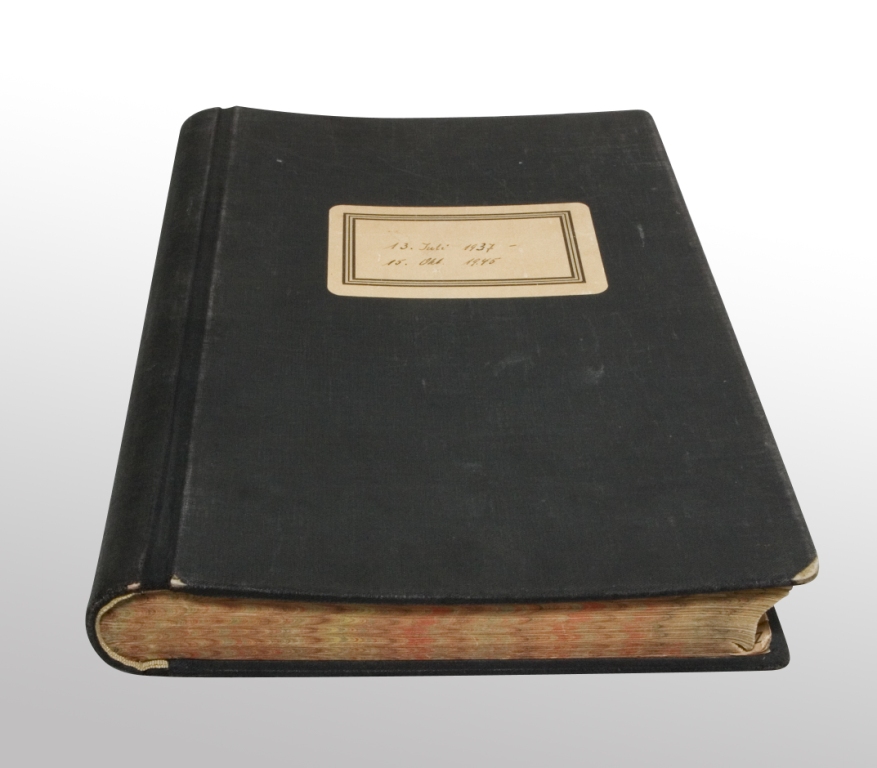

Supplement: Supplementary file 6 — Photo of the front page of Hugo Krayenbühl’s “Operationsbuch” containing his hand-written surgical records. From [33]. (JPEG 210 KB) [file 701_2017_3357_MOESM5_ESM.jpg]

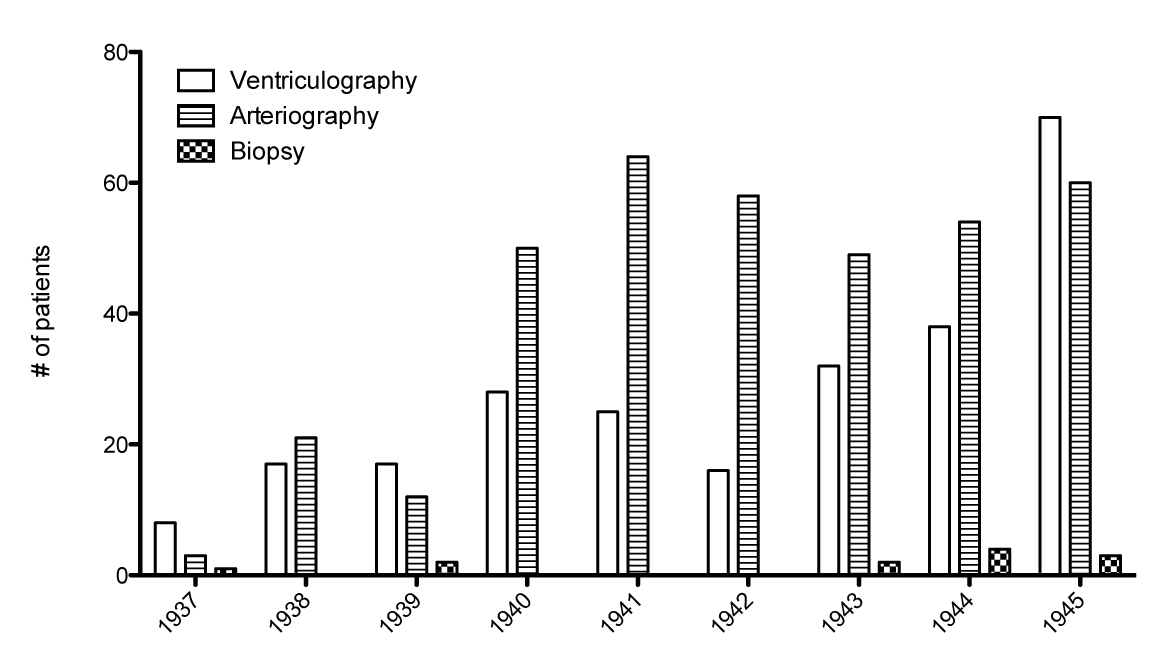

Supplement: Supplementary file 7 — Illustration of the rise in number of diagnostic (A) and therapeutic interventions (B) from the founding year of 1937 until 1945. The source of this data is the hand-written notes of Krayenbühl’s “Operationsbuch” (=surgical records) (adapted from [33]). “Spiller-Frazier” refers to the transection of the trigeminal nerve as described by Spiller and Frazier. [82] (JPEG 137 KB) [file 701_2017_3357_Fig12_ESM.jpg]

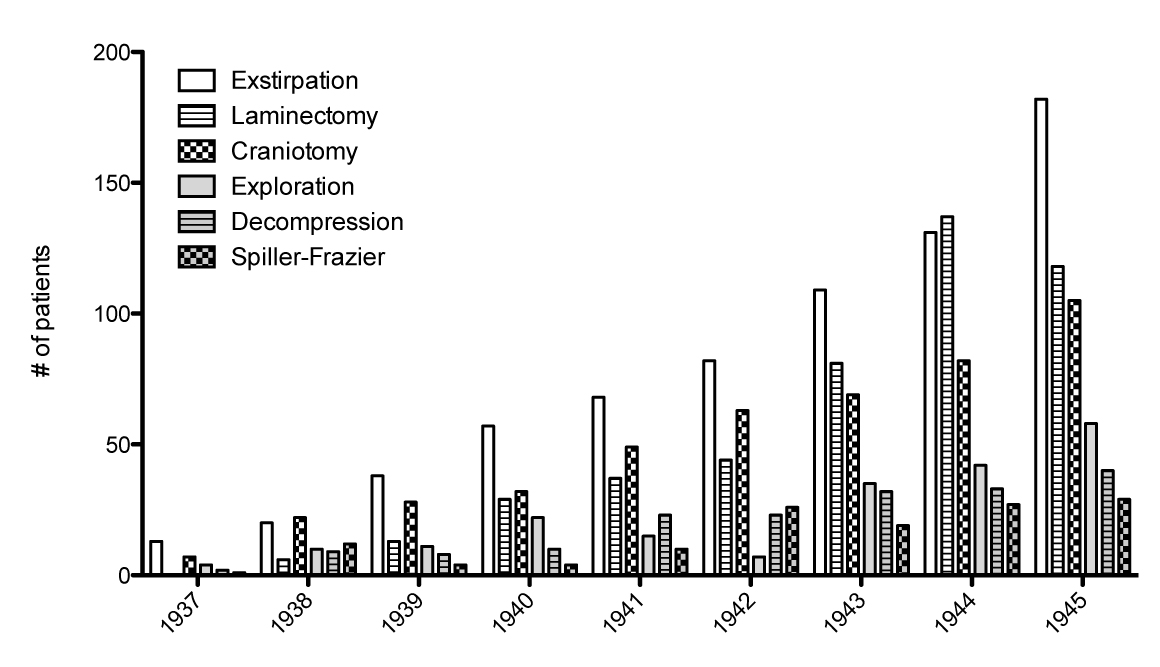

Supplement: Supplementary file 9 — (JPEG 196 KB) [file 701_2017_3357_Fig13_ESM.jpg]

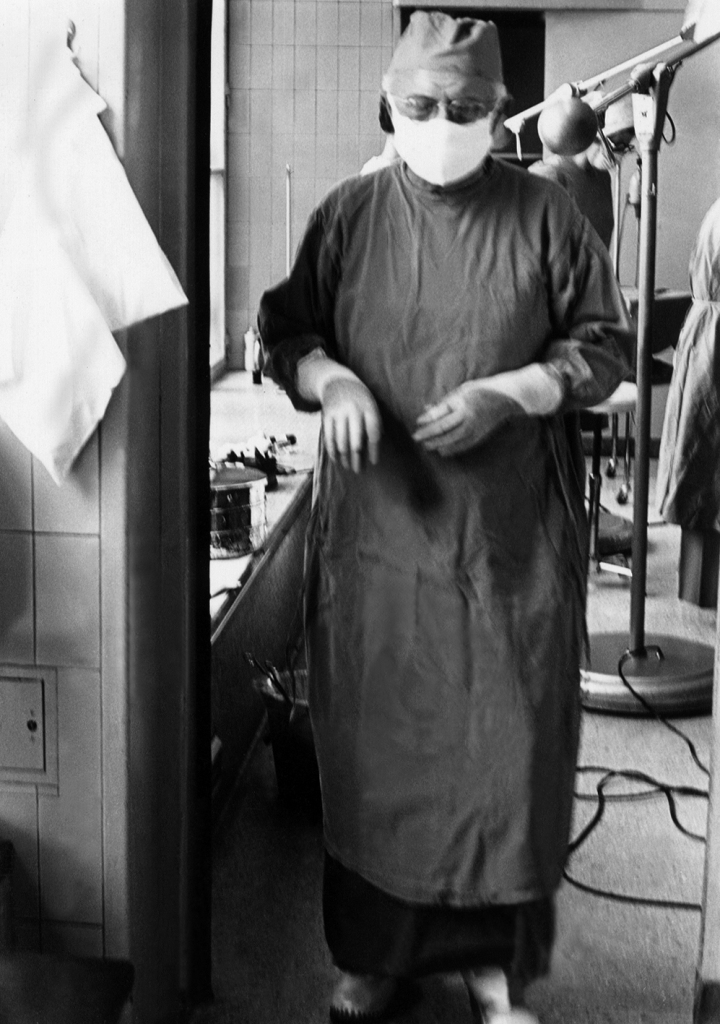

Supplement: Supplementary file 11 — Hugo Krayenbühl after his last surgery. Photo taken in 1973. From [33]. (JPEG 380 KB) [file 701_2017_3357_MOESM8_ESM.jpg]
